# Early Prefrontal Brain Responses to the Hedonic Quality of Emotional Words – A Simultaneous EEG and MEG Study

**DOI:** 10.1371/journal.pone.0070788

**Published:** 2013-08-05

**Authors:** Kati Keuper, Pienie Zwitserlood, Maimu A. Rehbein, Annuschka S. Eden, Inga Laeger, Markus Junghöfer, Peter Zwanzger, Christian Dobel

**Affiliations:** 1 Institute for Biomagnetism and Biosignalanalysis, University of Münster, Münster, Germany; 2 Institute of Psychology, University of Münster, Münster, Germany; 3 Otto Creutzfeldt Center for Cognitive and Behavioral Neuroscience, University of Münster, Münster, Germany; 4 Department of Psychiatry, University Hospital Münster, Münster, Germany; University of British Columbia, Canada

## Abstract

The hedonic meaning of words affects word recognition, as shown by behavioral, functional imaging, and event-related potential (ERP) studies. However, the spatiotemporal dynamics and cognitive functions behind are elusive, partly due to methodological limitations of previous studies. Here, we account for these difficulties by computing combined electro-magnetoencephalographic (EEG/MEG) source localization techniques. Participants covertly read emotionally high-arousing positive and negative nouns, while EEG and MEG were recorded simultaneously. Combined EEG/MEG current-density reconstructions for the P1 (80–120 ms), P2 (150–190 ms) and EPN component (200–300 ms) were computed using realistic individual head models, with a cortical constraint. Relative to negative words, the P1 to positive words predominantly involved language-related structures (left middle temporal and inferior frontal regions), and posterior structures related to directed attention (occipital and parietal regions). Effects shifted to the right hemisphere in the P2 component. By contrast, negative words received more activation in the P1 time-range only, recruiting prefrontal regions, including the anterior cingulate cortex (ACC). Effects in the EPN were not statistically significant. These findings show that different neuronal networks are active when positive versus negative words are processed. We account for these effects in terms of an “emotional tagging” of word forms during language acquisition. These tags then give rise to different processing strategies, including enhanced lexical processing of positive words and a very fast language-independent alert response to negative words. The valence-specific recruitment of different networks might underlie fast adaptive responses to both approach- and withdrawal-related stimuli, be they acquired or biological.

## Introduction

Emotional stimuli are perceptually processed with higher priority than neutral stimuli (for review, see [1]). This is reflected in faster detection times of emotional compared to neutral stimuli in behavioral paradigms (e.g., [2]), in enhanced amplitudes of event related potentials and magnetic fields (ERPs, ERMFs) in electro- and magnetoencephalography (EEG, MEG) (e.g., [3]–[7]), as well as in an enhanced blood oxygen level dependent response in the visual cortex (e.g. [8]). Thereby, one of the central sources of information for the identification of significant stimuli is their emotional value [1]. The motivational direction (approach vs. withdrawal) and strength of emotional stimuli can be characterized by two factors [9], [10]: their hedonic valence (pleasant vs. unpleasant) and their arousal (arousing vs. calm). These two factors are interdependent, such that pleasant or unpleasant stimuli usually evoke high arousal, while stimuli that are neutral are often rated as non-arousing [11].

Several ERP components relevant to human vision, such as the P1 (80–120 ms), the P2 (150–190 ms) and the Early Posterior Negativity (EPN, 120–300 ms) have been shown to be sensitive to emotional-neutral differentiations even if the organism is engaged in other activities (e.g. [12], [13]). For example, Carretié et al. [13], employed an oddball-task in EEG to investigate the spatiotemporal dynamics of automatic attention capture of positive, negative, and neutral deviant pictures in a stream of neutral standard pictures. The participants’ task was to mentally count the number of changes in color of the picture frame, i.e. their cognitive resources were involved in a task that was not related to the emotionality of the pictures. The data revealed enhanced activation in the anterior cingulate cortex (ACC) to negative pictures in the P1, whereas in the P2, both positive and negative pictures recruited this structure more than neutral ones. Such emotional attention mechanisms, also labeled motivated attention [14], have been postulated to ensure that an organism notices potentially revival relevant stimuli without effort. Interestingly, enhanced ERP-amplitudes have not only been found in response to emotional pictures (e.g. [4], [12], [13]) and faces (e.g. [5]), but also for stimuli that are entirely symbolic and have acquired their (emotional) meaning by learning, such as gestures [15] and emotional words ([16]–[22], for reviews, see [23], [24]). Studies on emotional word processing have primarily focused on differences in perception between emotionally arousing and neutral words, usually reporting differences in the EPN and the P1 [16]–[20], [22], [25]. By contrast, the time course of valence effects – when arousal was controlled for – is less clear: Whereas some studies have suggested that in words, valence is processed prior to arousal [16], [26], that is as early as the P1, others have either reported later valence effects [17], [27]–[30], or none at all (e.g., [19], [20]). Similarly, the direction of valence effects in words remains poorly understood. While for pictures, there seems to be a processing advantage for negative compared to (equally arousing) positive stimuli (e.g., [31]; for review see [32]), words have often shown the reverse pattern, with an advantage for positive over negative words – again keeping arousal constant. This positivity bias in words has been substantiated by superior behavioral performance (e.g. [30], [33]–[36], but see [37], [38]), by more pronounced ERPs (e.g. [16], [17], [27]–[30], [34], but see [22]) and by a stronger hemodynamic activation in regions supporting semantic retrieval (bilateral middle temporal and superior frontal gyrus) [36] as well as the amygdala [17]. As for non-symbolic emotional stimuli, functional magnetic resonance imaging (fMRI) studies have revealed higher amygdala activity for arousing negative and/or positive words than for neutral words (e.g., [17], [39]–[44]) suggesting that the amygdala, a key structure in emotional processing (e.g., [45]), preferentially processes emotional arousal. In contrast, frontal structures have often been linked to the processing of valence [41], [43], [44], [46]. Regarding valence effects in prefrontal structures, there are two prevailing views. The hypothesis of hemispheric asymmetry ([47], [48], for a critical review see [49]) claims that the left hemisphere, specifically the left PFC, is more involved in positive (approach-related) emotions, whereas the right hemisphere is dominant during negative (withdrawal-related) emotions. Support for this hypothesis stems mainly from electrophysiological and lesion studies, but a valence-specific hemispheric asymmetry is not readily found in fMRI studies (e.g. [46], [50]–[53]; but see [54]–[56]). Imaging data gave rise to a second proposal, according to which lateral orbital PFC regions are preferentially activated by negative stimuli, whereas ventromedial PFC regions are more sensitive to positive stimuli (e.g. [46], [52]). Yet, the time course of prefrontal effects of valence remains elusive. There is some evidence from EEG and MEG that high-arousing negative stimuli (e.g. negative pictures and aversively conditioned stimuli) recruit prefrontal structures, such as the anterior cingulate cortex (ACC) [13] and the orbital and dorsal prefrontal cortex (for review, see [57]) very rapidly, even before 120 ms. However, in verbal stimuli, there is so far no evidence for early valence-specific prefrontal activation (for review, see [58]), nor for the validity of any of the above hypotheses.

There are several possibilities to account for this: First, right hemispheric and/or frontal valence effects for symbolic word stimuli may well depend on access to their (hedonic) meaning, involving the left hemisphere. In fact, the so-called time course hypothesis implies a shift of activation from the left to the right hemisphere while semantic information is processed [59], [60]. A similar line of argumentation might apply to effects of arousal. In line with these considerations, all studies employing source reconstruction methods have localized arousal effects in posterior left temporal, language-related [19], [20], [25], or occipital [18], but never in prefrontal regions. Second, valence effects in words might be weak and could therefore easily be missed by common ERP recordings. Most previous studies have applied conventional single-modality (EEG) methodologies [18], [20], [25] with the exception of [19], the companion paper to the current study, in which we analyzed the same data as presented here, but with a different goal: In this study [19], we focused on the evaluation of a combined EEG-MEG source reconstruction methodology and applied it on arousal effects which have proved quite robust in words. Combined EEG-MEG recordings are able to uncover and localize activity even in rather deep structures such as the posterior cingulate [61]. We were able to find such activity in the EPN time window (200–300 ms) in which emotional were distinguished from neutral words. To give as much credibility to our results as possible, we employed an extremely conservative approach by using (1) a significance criterion of P = 0.001 and (2) by determining a cluster extend threshold at a level of P = 0.001. Even with this very conservative approach, we confirmed previous EEG work with regard to the time course for distinguishing emotional from neutral words (e.g. [20]) and previous fMRI work with regard to the localization of these effects [62]. This not only permitted a high confidence in the results, but also in the employed source reconstruction methodology. Overall, in ([19], see table S2 in tables S1), we reported stronger neural responses to high-arousing (positive and negative) nouns compared to low-arousing neutral nouns in occipital, parietal, and posterior cingulate regions during the EPN and in the left middle temporal lobe during the P1 (80–120 ms), as well as convergent behavioral effects. In line with brain responses, participants remembered more emotional than neutral words in an unannounced free recall. Confirming a positivity bias in response to positive words, we also observed a tendency towards a better memory for positive compared to negative words (P = 0.064), but no neurophysiological valence-specific correlates. While the extremely conservative approach used in [19] did not target valence effects, we here ask if such effects can be uncovered with a more common and less conservative approach in the same data set. If it is true that – independently of the task –valence is processed simultaneously or even before arousal, we expect to find valence effects as early as the P1 [16], [26], possibly with an involvement of prefrontal structures [13]. We ask how positive and negative words are differentiated with respect to their time course and localization. If the reported processing advantage for positive words is best explained by accelerated/enhanced lexical processing, we predict enhanced activity for these words in the left temporal lobe, crucial for lexico-semantic processes. A next question is whether prefrontal hemispheric asymmetries play a role in valence processing in words, and if so, what is the time course? To approach these questions, we focus on three emotion-sensitive intervals of interest: the P1, the P2, and the EPN. We think that our methodological approach of combined EEG/MEG source reconstruction on the basis of individual realistic head models will be more sensitive to valence effects than single modality recordings, due to its better sensitivity [63] and source localization accuracy [64], [65] in prefrontal [66] and temporal cortex areas [67] – regions of specific importance for valence and language processing, respectively (for details, see [19]). To anticipate our results very briefly, we found valence effects as early as 100 ms in cortical networks including prefrontal and language related areas in the P1 and P2. However, already at this point, we would like to point out that compared to arousal effects, valence effects seem to be much weaker and harder to uncover.

## Materials and Methods

### Participants

Twenty healthy, right-handed German native speakers (aged 21–31 years, eleven female) were selected from a database of the Institute for Biomagnetism and Biosignalanalysis in Münster, Germany. Inclusion criteria were right-handedness, no current or former severe neurological or psychiatric disorder and normal or corrected to normal vision. All participants were familiar with the MEG and EEG testing procedure and were financially compensated with nine Euros per hour. Due to strong and continuous artifacts in the EEG-recordings, two participants were excluded from the analysis.

### Ethics Statement

This study was approved by the Ethics Committee of the Medical Faculty of the University of Münster (2009-392-f-S). All participants provided their written informed consent to participate in this study.

### Stimuli

Stimuli consisted of 180 German high-arousing positive (e.g. *love*), high-arousing negative (e.g. *pain*) and low-arousing neutral (e.g. *month*) nouns (taken from [20]). Arousal and valence were assessed by a rating with 45 students, using the Self-Assessment Manikin [11]. Arousal levels of words with a positive or negative valence did not differ, but exceeded those of neutral nouns. The three word categories were matched for length, frequency of use (based on the CELEX database; [68]), concreteness and neighborhood size (as obtained from the dlex database under http://dlexdb.de/) (for more information and statistical values, see [19], table S1 in tables S1).

### Procedure

The individual EEG-electrode positions for each subject were digitized using a 3D tracking device (Polhemus, Colchester). Participants were then seated in the magnetically shielded, sound-attenuated, and dimly lit MEG chamber. To monitor the head position in the MEG scanner, three landmark coils (two auditory channels and the nasion) were recorded by means of the Polhemus 3Space® Fasttrak. Head motion during the MEG/EEG measurement was below 0.5 cm for all participants.

Participants were instructed to covertly read the words presented visually in black characters on a light grey screen, with a viewing distance of 90 cm and an average visual angle of 3.93° (center to edge, SD = 1.24°). Each participant was shown five repetitions of differently randomized RSVP (rapid serial visual presentation) sequences. Each sequence consisted of a stream of 180 words (60 positive, 60 negative, 60 neutral) that were each presented for 1,000 ms, without inter-stimulus intervals (1 Hz presentation). Transitional probabilities between the three conditions were kept equal, and sequence orders were counterbalanced across subjects. Immediately after the measurement, participants completed an unannounced free-recall task in which they were asked to recall as many words as possible.

### Neurophysiological Recording and Data Analysis

Neurophysiological signals from 275 MEG sensors, 80 EEG electrodes and an electrocardiogram (ECG) were recorded simultaneously with a sampling frequency of 600 Hz and an on-line low-pass filter of 150 Hz, by means of a whole-head MEG/EEG-system (Omega 275, CTF, VSM MedTech Ltd.) with first-order axial SQUID gradiometers (2 cm diameter, 5 cm baseline, 2.2 cm average inter-sensor spacing). The 80 EEG electrodes, including six ocular electrodes (EOG), were mounted on a flexible MEG compatible lycra electrocap (easycap, Falk Minow Services, Munich Germany), placed in accordance with the extended version of the international 10–10 system and referenced to FCz during recording. All electrode impedances were kept below 8 kΩ. ECG was recorded by means of electrodes attached to the subjects’ right cervix and left costal arch.

### Preprocessing of EEG-MEG-Data

**Figure 1:**
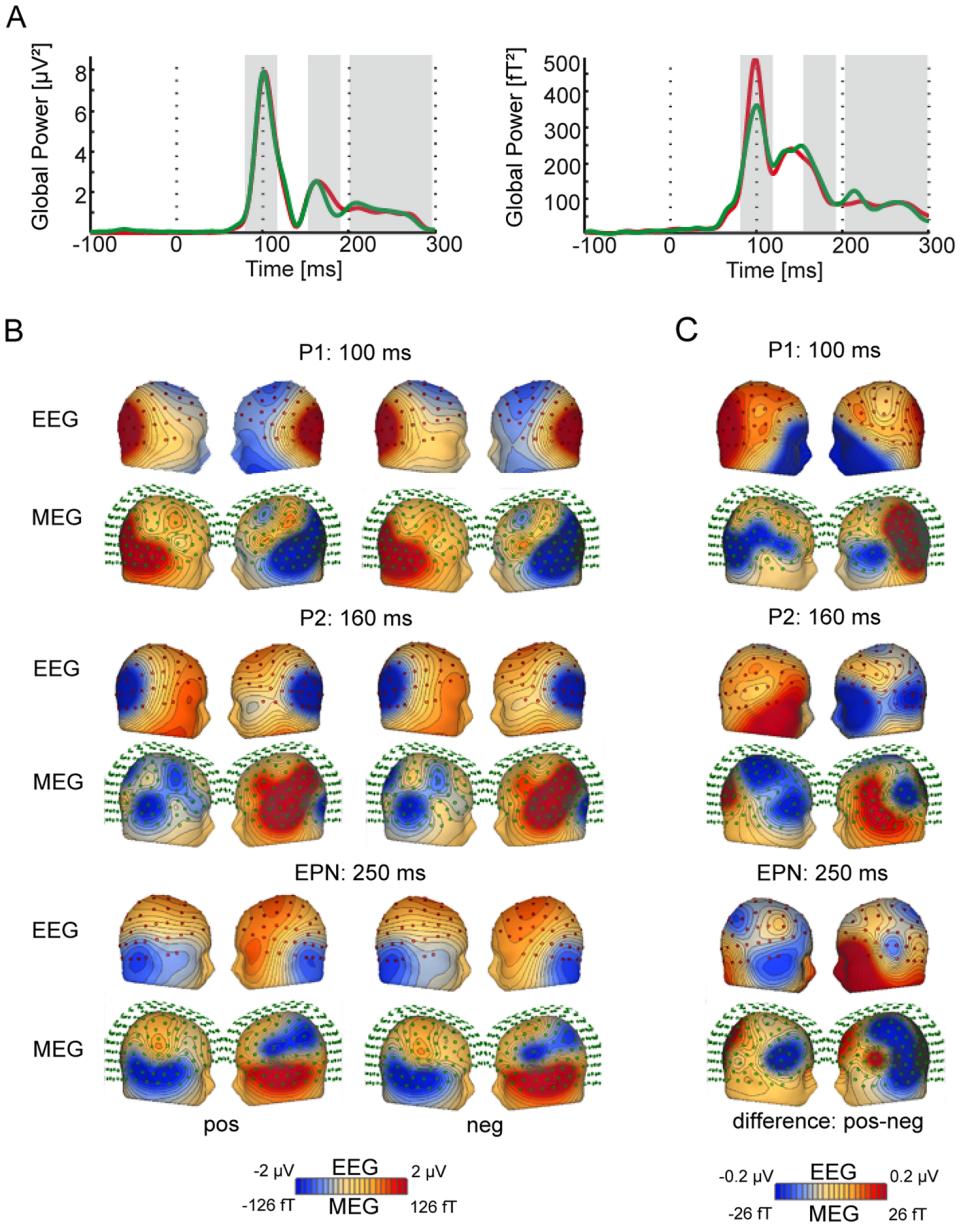
Event-related potentials and fields in response to positive and negative words. (**A**) Evoked potentials/fields (ERPs/ERMFs) during reading of positive (green) and negative (red) words. The graph displays the GP across all sensors for the EEG (left) and the MEG (right). The critical time intervals (P1, P2 and EPN) for the CDRs are shaded in gray. (**B**) Scalp/field distribution of sensor space activity in EEG and MEG, depicted separately for the positive (left) and negative (right) condition for three intervals of interest (top: P1, middle: P2, bottom: EPN). The red dots indicate EEG-electrodes (upper row), the green circles represent MEG sensors (lower row). Cooler colors indicate more negative-going potentials/fields, whereas warmer colors display more positive-going potentials/fields. (**C**) Scalp/field distribution of the scalp/field potential difference (activation for positive minus activation for negative words) in EEG and MEG, depicted for the three intervals of interest (top: P1, middle: P2, bottom: EPN).

Offline preprocessing was done with Brain Electrical Source Analysis (BESA 5.3) software. EEG and MEG recordings were filtered using a 40 Hz low-pass and a 1 Hz high-pass filter. Correlates of ocular activity were corrected by applying an adaptive artifact-correction method [69]. EEG electrodes with sustained artifact contamination were interpolated, if fewer than six non-adjacent sensors were affected. Otherwise, the participant was excluded from further analyses. The averaging epoch for each trial lasted from 200 ms before to 600 ms after stimulus onset. To avoid phasic artifact contamination, trials exceeding a magnetic field strength of 3000 pT in the MEG or a potential of 120 uV in the EEG in any sensor were excluded from subsequent analysis. The number of excluded trials did not differ across emotional-word conditions (F (2,34) = 1.416, P = .257;.255 (Greenhouse-Geisser corrected). For each condition, averages were computed and subsequently down-sampled to 250 Hz. Critical time intervals for the P1 (80–120 ms), the P2 (150–190 ms) and the EPN (200–300 ms) were determined a priori on the basis of the current literature [13], [20], [25]. The global power (GP) amplitudes of EEG and MEG sensor space signals served to verify the appropriateness of these intervals in this dataset (see figure 1, see also [19]).

### Current Density Reconstruction on the Basis of Individual Boundary Element Models

As *volume conductor models*, we used three-compartment boundary element models (BEM) comprising skin, skull, and brain that were generated on the basis of individual T1-weighted anatomical scans recorded with a 3-Tesla Magnet Resonance Imaging (MRI) Scanner (Gyroscan Intera T30, Philips, Amsterdam, Netherlands) prior to the experimental session. Preprocessing steps of these images are described in detail in the companion paper [19]. Using the CURRY software package (version 6; Compumedics Germany GmbH, Hamburg), these MRI-scans and the individual EEG and MEG sensor positions were co-registered by aligning the anatomical landmarks (nasion, left and right preauricular points). For the individual BEMs, a mesh size of 9, 8 and 6 mm and conductivity values of 0.33, 0.0042 and 0.33 S/m were chosen for the skin, skull and brain boundary elements, respectively [70]. A source model using a cortical triangle mesh with 3 mm triangle side length was built on the basis of the gray matter segmentation. This model contained the hemispheric gap but was constrained to the cortex by excluding the brainstem and the cerebellum. Depending on the individual brain anatomies, this procedure resulted on average in 14863 (SD = 1911) dipole locations. We computed combined (EEG and MEG) *current-density reconstructions (CDR)* using the Minimum Norm Least Squares (MNLS, L2-Norm) method (see [71]) - an inverse method that allows the reconstruction of distributed neural sources without requiring a-priori assumptions regarding the number and possible locations of underlying neural generators. To calculate the MNLS estimate, the pseudo-inverse of the so-called lead-field matrix (which describes the sensitivity of each sensor to the sources) was multiplied with the averaged recorded data. The Tichonov regularization parameter lambda, needed for the calculation of the pseudo-inverse, was based on an estimation of individual noise levels within a pre-defined baseline interval, ranging from 150 to 50 ms before stimulus onset. The individual noise level was computed from the average of standard deviations of all channels separately for EEG (mean across subjects: M = 0.33, SD = 0.09) and MEG (mean across subjects: M = 8.40fT, SD = 1.84). To address different noise levels between EEG and MEG, a whitening procedure on the basis of the noise variance estimated from this interval was used. Note that differences between experimental conditions of ERPs and ERMFs within this interval cancel out, due to experimentally balanced transitional probabilities. A square-root compensation was applied [72] to correct for the undesired depth dependency of L2-minimum norm solutions. Finally, mean L2-norm solutions for the P1 (80–120 ms), P2 (150–190 ms), and the EPN (200–300 ms) were averaged separately for each condition and subject. In order to eliminate individual differences in brain structure for the statistical analysis, individual CDRs were normalized to a standard space, using the SPM8 software package (for more details on the normalization procedure, see [73]). CDRs were masked and smoothed with an adapted template of the cerebral cortex restricted to gray matter.

Voxel-wise one-way within-subject Analyses of Variance (ANOVA) were carried out separately for the CDRs of the time intervals of interest, using the SPM8 software package, to test for differential neural generator activation across different experimental conditions.

To control for false positives due to multiple testing, we used the Alphasim [74] implementation in REST [75] to determine a spatial cluster-extend threshold. This method yields an estimation of the probability for a cluster to occur on the basis of 5,000 Monte Carlo simulations. Thereby, a cluster was defined as a group of voxels with P-values of ≤0.05 that were separated by no more than one voxel width. We estimated the cluster extend threshold on the basis of all voxels that were considered possible sources of the CDRs, by applying the mask used on the CDRs. This procedure yielded an empirically determined minimum cluster size of 774 voxels for a cluster P-value of 0.05. Note that the choice of a significance criterion of P≤0.05 as compared to P≤0.001 in the companion study [19] on the one hand reduces the beta error (i.e. misses), but on the other hand makes an alpha error (i.e. false positives) more likely.

## Results

### Recall Performance

Effects of the emotionality of the words on the recall performance, including effects of arousal, are described in detail in [19]. Here, we focus on effects of valence. Participants correctly recalled 11.9 positive (SD = 5.5) and 9.9 negative (SD = 6.2) nouns. Post hoc comparisons (Bonferroni corrected) comparing positive and negative words revealed a trend towards a better memory of positive compared to negative words (t(17) = 2.1; P = .064 (two tailed)).

### ERP Data in Sensor Space

The time course of activity in sensor space is shown separately for EEG and MEG in figure 1 (Panel A). In EEG, the global powers of the amplitudes for the positive (green) and negative (red) condition are quite similar in the P1, and the EPN, but reveal a difference in the P2. By contrast, global powers of MEG amplitudes suggest differences in the P1, and to a smaller degree, in the P2 and during the onset of the EPN. Note that in sensor space, high global power amplitudes may be induced by both positive and negative values. Therefore the direction of effects cannot be interpreted directly. The electric potential and magnetic field distributions during the peaks of the P1 (100 ms), P2 (160 ms), and EPN (250 ms), are depicted separately for the negative and positive emotional condition in Panel B. Panel C displays the potential and field differences between the positive and negative condition plotted on a head model. For the P1, we observe that positive compared to negative words elicit a relative positivity (red) over posterior regions and a stronger negativity (blue) over anterior regions in EEG. The corresponding MEG distribution shows multiple and widely distributed areas with ingoing and outgoing difference fields. The strongest enhanced outgoing field (positive, red) is localized over left temporo-occipital regions, smaller outgoing difference fields are observed over bilateral frontal areas. The complexity of the MEG distribution suggests several simultaneously active neural generators. During the P2 (160 ms), the EEG uncovers a stronger right-lateralized anterior positivity and a stronger left lateralized anterior negativity for positive compared to negative words. The corresponding MEG difference field distribution again shows widely distributed areas, with the strongest outgoing difference field in left temporo-frontal and right occipital areas. For the EPN (250 ms), the difference map between the positive and negative conditions again suggests simultaneous activity of various areas. The EEG shows stronger relative positivities for positive word processing over left fronto-temporal regions and negativities over right frontal, temporal and parietal regions. The MEG also reveals widely distributed areas, with the strongest outgoing difference fields over left frontal and right occipito-parietal areas.

### Combined EEG-MEG Current-density Reconstructions

**Figure 2:**
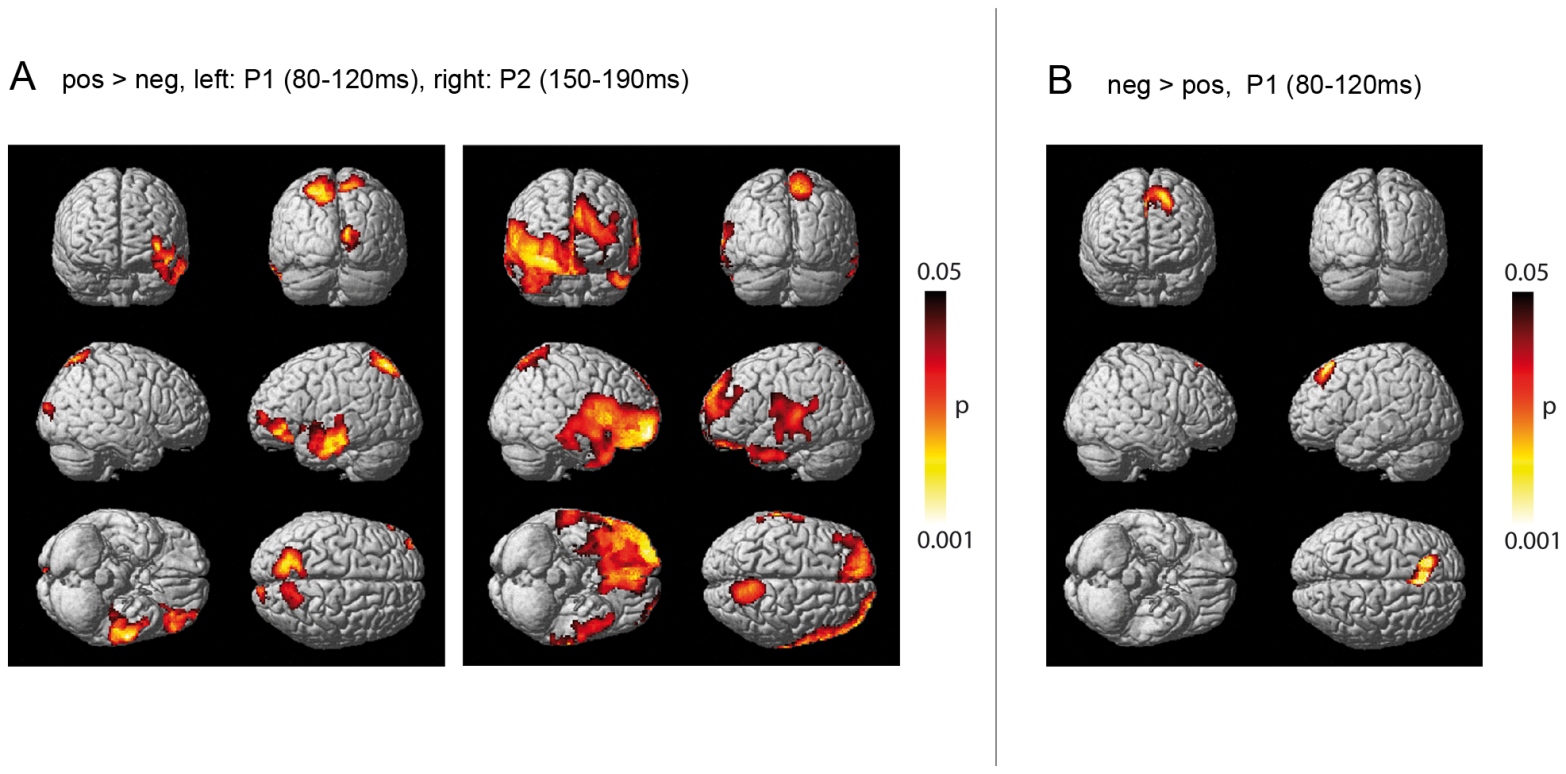
Neural generators of valence effects in the P1 and P2 time interval. (**A**) Cortical regions differential activation patterns for positive (pos) compared to negative (neg) words in the P1 (left) and the P2 (right) (**B**) Cortical regions displaying enhanced activation to negative compared to positive words in the P1. No other contrast met our significance criteria. All images were thresholded using a voxel-wise statistical height threshold of (P<.05, Alphasim corrected at k = 744). Functional images are superimposed on a standard (SPM: render_single_subj):

In the following, we will present results from the statistical analyses of the combined EEG-MEG-current density reconstructions of the P1 (80–120 ms), the P2 (150–190 ms) and the EPN (200–300 ms) time intervals. All analyses were based on planned contrasts (positive>negative and negative >positive condition) in a one-way ANOVA (within subject), with the significance level set at P<.05. Figure 2 shows all clusters showing significant activation differences between positive and negative words that survived the Alphasim correction. The corresponding peak T values for each cluster, as well as peak coordinate labels and cluster sizes are presented in table 1. Note that the labels mentioned in table 1 refer to the peaks of each cluster. However, due to large cluster sizes, the regions showing significant differences between conditions are relatively broad and extend to other structures. This can be seen in figure 2 and will be described in detail in the text.

**Table 1:** Regions of activation differences between positive (pos) and negative (neg) words in the P1 (80–120 ms) and in the P2 (150–190 ms) interval.

Time window	Brain Region (peak)	BA	Cluster size	MNI coordinates of local maximum			T
				X	Y	Z	
**pos>neg**							
P1∶80–120 ms	Temporal_Mid_L		3871	−52	−20	−19	2.83
	Parietal_Sup_L		1783	−26	−70	61	2.74
	Calcarine_R		832	10	−94	11	2.45
P2∶150–190 ms:	Frontal_Mid_Orb_R	11, 10	21259	30	60	−11	3.86
	Temporal_Sup_L	42	2051	−68	−27	7	3.29
	Parietal_Sup_R		1502	14	−70	65	3.06
	Temporal_Inf_L		952	−54	8	39	2.51
**neg >pos**							
P1∶80–120 ms	Frontal_Sup_Medial_L	32	1465	−4	24	39	2.53

x, y, z = coordinates according to MNI stereotactic space (Brain Region according to AAL-Atlas [106], L = left, B = bilateral, R = right, sup = superior, mid = middle, inf = inferior), T = peak T value for the respective contrast of the valences, BA = approximate Brodmann’s area, Cluster size in voxels, P>.05 (Alphasim corrected).

The current density reconstructions reveal enhanced activation for positive compared to negative words (see figure 2, Panel A) in the P1 and in the P2 interval. When inspecting the GP plots in sensor space (Figure 1A), it appears that negative words cause more activation than positive words. However, since high amplitudes in GP include both positive and negative values, the sensor space activation maps cannot easily be interpreted in terms of intensity of the underlying neuronal signal. In fact, the direction of effects may be quite different in source space. In the EPN, this contrast did not survive the Alphasim correction. In the P1, three clusters display higher estimated neural activity for the positive compared to the negative condition (pos>neg). The first cluster peaks in the left middle temporal lobe, and expands to the left inferior and middle frontal lobe, both of which are parts of the prefrontal cortex (PFC). The second cluster is located in the bilateral parietal lobe and precuneus, with a peak in the left superior parietal lobe. The third cluster is located in the occipital lobe and includes the right calcarine, the right superior occipital lobe as well as parts of the right lingual lobe and the middle occipital lobe. Similarly to the P1, the P2 reveals several clusters with enhanced activity for positive over negative words. The first cluster comprises the bilateral frontal and temporal lobe, predominantly in the right hemisphere. It peaks in the right middle orbital frontal cortex (BA 11) and extends to the ACC, both of which belong to the PFC. A second cluster has its peak in the left superior temporal lobe (BA 42), and extends to the middle temporal lobe and the pre- and postcentral gyri. Activity in the right superior parietal (cluster 3) and the left inferior temporal lobes (cluster 4) is also larger for positive than for negative words.

In contrast to this temporally and spatially widely distributed positivity bias, activation for negative words exceeded positive words only in the P1 time interval, and only in one significant cluster positioned at the left superior medial frontal lobe. This prefrontal cluster extends to the right ACC and the left middle frontal lobe.

Although EPN effects did not survive Alphasim correction, we wish to document which clusters show valence effects, given that the EPN is one of the most prominent emotion-sensitive components (see table 2).

**Table 2:** Regions of activation differences between positive (pos) and negative (neg) words in the EPN (200–300 ms) with a cluster size of k>30.

Time window	Brain Region	BA	Cluster size	MNI Coordinates of local maximum			T
				X	Y	Z	
**pos>neg**	SupraMarginal_R	40, 2, 34, 1, 42	243	68	−62	23	2.37
	Precentral_L	6, 8, 4	175	−54	4	47	2.17
	Frontal_Sup_L	10, 9	461	−16	60	29	2.08
	Angular_R	39	109	50	−56	21	2.03
	SupraMarginal_R	40	107	50	−42	31	1.99
	Precuneus_L		39	0	−58	71	1.98
	Occipital_Inf_R	19, 18	54	48	−82	−5	1.97
	Precuneus_L	7	119	−8	−72	63	1.94
**neg >pos**	Cuneus_R	7, 19	404	16	−78	35	2.28
	Frontal_Mid_R	8	88	42	24	51	2.10
	Precentral_R	6,9	35	40	2	41	1.83

x, y, z = coordinates according to MNI stereotactic space (Brain Region according to AAL-Atlas [106], L = left, B = bilateral, R = right, sup = superior, mid = middle, inf = inferior), T = peak T value for the respective contrast of the valences, BA = approximate Brodmann’s area, Cluster size in voxels, P>.05 (uncorrected).

## Discussion

This study investigated the spatiotemporal brain dynamics of spontaneous valence processing of high-arousing positive versus high-arousing negative nouns, visually presented for silent reading. In order to optimize the temporal and spatial resolution and to obtain the full neurophysiological signal, a combined EEG/MEG source reconstruction was performed on the basis of realistic head models, for three intervals of interest: The P1 (80–120 ms), the P2 (150–190 ms) and the EPN (200–300 ms).

There were four main findings: First, positive and negative words at least partly recruited different brain regions early in the processing stream. This supports previous findings that positive and negative words are dissociated as early as the P1 [16], [26]. In particular, relative to negative words, positive words elicited stronger neuronal activity (1) in left temporal and prefrontal, bilateral parietal, and occipital regions during the P1 and (2) in the left temporal lobe, distributed bilateral fronto-temporal areas, including prefrontal regions such as the ACC and the orbital lobe, and in the right superior parietal lobe during the P2. In contrast, negative words caused enhanced activity in the P1 only. This effect was located in a prefrontal cluster comprising the left superior medial frontal lobe, the ACC and the left middle frontal lobe. These early valence specific effects presumably reflect an “intrinsic pleasantness” check, as proposed by the component process model of emotion [76]. Second, in line with the assumption of a positivity bias (e.g. [30], [33]–[36], but see [37], [38]), positive words were at trend better recalled and elicited stronger neural responses in cortical networks associated with lexical processing (left temporal and inferior frontal regions; see [36]) than negative ones. Importantly, our results add temporal detail by linking these effects to the P1 and P2. Third, prefrontal structures were involved in the generation of valence effects in the P1 and the P2. This provides support for the hypothesis that prefrontal structures are particularly important during the processing of valence [41], [43], [44], [46] early in the processing stream [13]. Fourth, from the fact that we did not find valence effects in a previous extremely conservative analysis of the same data [19], we can infer that these effects are less pronounced than arousal effects. Neither the contrast Neg >Pos in the P2 interval, nor any contrast in the EPN interval survived the Alphasim correction.

In the following, we first address the relevance of our effects for the claim of speeded lexical processes in response to emotional compared to neutral words, with faster access to its subcomponents, e.g. to word forms and meanings. (cf. [19], [25]). We particularly focus on the assumed lexical advantage for positive over negative words (e.g. [25], [36]), by discussing effects in language related regions (left temporal and inferior frontal) that display more activity in response to positive than negative words during the P1 and the P2. However, when taking into account *all* cortical structures that are differentially activated by positive and negative words, it is obvious that the observed effects do not reside in lexical processing only. Thus, second, we will discuss regions that are commonly not found in studies on lexical processing. We will thereby evaluate the hypothesis that they represent valence-specific “tags” of the word form [19], generated on the basis of previous learning experiences. For example, if a pre-lingual child is approached by a *butterfly* vs. a *wasp* the mother’s reaction (“Look there is a butterfly” vs. “Be careful, there is a wasp”) will cause different learning experiences. Whereas the child will direct its attention towards the *butterfly*, it will associate the *wasp* with a state of alert, probably without an elaborate perceptual analysis, but with a focus on a potential exit strategy. The idea of an “emotional tagging” assumes that these perceptually and physiologically different mechanisms are represented in the mapping between the word form (*butterfly* vs. *wasp*) and a (positive vs. negative) meaning. Thus, if valence effects in response to words reflect a (valence-specific) tagging during language acquisition, one would expect to find similar regions to be active in words as in neutral stimuli that have been affectively conditioned, or in non-symbolic stimuli.

### Regions Displaying More Activity to Positive Compared to Negative Words

#### Lexical effects in the left temporal and inferior frontal lobe in the P1 and the P2

The most pronounced cluster displaying enhanced activity for positive over negative words peaks in the left middle temporal lobe, and expands to inferior and middle frontal regions. Both the left middle temporal lobe, which houses parts of the mental lexicon (e.g. [77]), and the left inferior frontal cortex have often been linked to different language-related demands, including lexical and semantic processing (e.g. [78]–[81]). The middle temporal lobe has also been shown to generate effects of arousing compared to neutral words in the P1 time range [19], [25]. The locus and timing of these effects support the view that the emotional quality of words is reflected in their enhanced lexical processing. Here, we add that the middle temporal lobe also subserves valence effects, with enhanced activity for positive relative to negative words. Together with data from the companion study [19], showing arousal effects in the left middle temporal lobe, these findings suggest that arousal and valence effects are manifest at the same time in the same brain areas. One interpretation is that arousal differentially influences lexical processing of positive and negative words [25], however it is also possible that arousal and valence effects simply add up in this region.

As we discussed in detail elsewhere (e.g. [19]), there is ample evidence from psycholinguistic studies that lexical processing starts as early as 100 ms after stimulus onset ([82]; see also [83]–[87]). Combined EEG-MEG data [83] have localized effects of the lexical status (word-pseudo-word contrast) to the left middle temporal lobe, though slightly later than our P1 effect. Thus, both the topography and the timing of our effect fit well with the assumption of an early lexical advantage for positive over negative words. This might reflect accelerated access to lexical representations (lexical access), an interpretation that is supported by studies comparing lexical decision latencies to differently valenced words: When other lexical variables (e.g. frequency, word length) were controlled for, positive words were reacted to faster, and more accurately than negative words (e.g., [30], [34]–[36], [38], [88], [89]).

Similar to the left temporal and inferior frontal effects observed in the P1, the P2 also revealed more activity for positive than for negative words in the left temporal lobe (left middle and superior temporal lobe, left inferior temporal lobe). This may indicate continuing lexical and semantic processing, which is commonly assumed to proceed in parallel (e.g. [84], [90]). In addition to the left temporal effect, the P2 revealed a broad cluster comprising the bilateral frontal lobe. Note that – compared to the left hemispheric dominance during the P1– effects in the P2 were less lateralized. If we assume that the left temporo-frontal effects in the P1 reflect lexical and/or semantic processes, this finding is in line with the time-course hypothesis, which implies a shift of activation from the left to the right hemisphere while semantic information is processed (e.g. [59], [60], see also [58]). Thus, the left temporal initiation of the valence effect (Pos>Neg), as well as its development over time support the idea of an early lexical advantage for positive over negative words.

#### Non-lexical valence effects in the P1 and the P2

Not only left lateralized, language-related brain regions showed enhanced activity to positive compared to negative words. Additionally, we found right-lateralized occipital and bilateral parietal regions to display more activity for positive words during the P1. The right superior parietal lobe remained more active in response to positive words, until the P2. Similar localizations of valence effects (Pos>Neg) have also been reported in fMRI studies (e.g. [36]). Both occipital and parietal effects might be attributed to additional attention for positive stimuli in the visual association cortex (VAC), a system in which attention is clearly engaged in information processing (cf. [91]), as predicted in our example (“butterfly” vs. “wasp”). There are some hints from conditioning studies, in which multiple auditory or visual conditioned stimuli (CS) were paired with unconditioned stimuli (US) (MultiCS-conditioning, see [92]–[94]) that point towards enhanced activity for positive over negative stimuli in the parietal lobe. To our knowledge there is only one conditioning study which, in addition to negative stimuli, also used positive US [93]. Although Bröckelmann et al. [93] did not specifically focus on the differentiation between positively-, and negatively-conditioned stimuli, cortical activation over (right hemispheric) parietal sites appears to be stronger for positive compared to negative items early in the processing stream [93]. Additionally, there is evidence for enhanced processing of positive compared to negative stimuli in the VAC even in non-symbolic stimuli [95]. Similar to our effects, this study reported enhanced occipital voltage and dipole strength for positive compared to negative emotional pictures between 120 and 150 ms after stimulus presentation. In the light of these findings, the P1 effect in the VAC observed in our study might in fact reflect a valence-specific tagging of the word form during acquisition.

Still, this finding does not explain, why processing of positive stimuli is also enhanced in language-related structures. What is the relationship between additional attention in the VAC, and enhanced lexical processing in left temporal regions? What is their functional interplay? One explanation might be that positive valence per se enhances lexical access. However, it is also possible that additional attention allocation in the VAC interacts with other variables that are known to affect lexical access. We controlled for many lexical variables (including frequency, imageability, word length), but recently, it was shown that positive words are usually rated as more familiar than negative words [96], even when word frequency was controlled for. In the present study, we did not control for familiarity, thus valence and familiarity might be confounded. However, even in this case, one might speculate that the additional VAC-activation to positive words is responsible for the higher familiarity values of positive words, *because* this activation secures enhanced information processing (cf. [91]), and might thus lead to higher familiarity ratings. In this case, the often reported lexical advantage of positive words would be mediated by familiarity.

In addition to the effects in the VAC and in language-related structures, bilateral fronto-temporal areas were more strongly activated for positive than for negative words in the P2, with a peak in the right middle orbitofrontal cortex. The orbitofrontal cortex is involved in a variety of emotion-related functions, including the representation of affective value of reinforcers and punishment (for review, see [97]). A meta-analysis on the functional anatomy of this region reveals a functional division of this structure in which, in line with our data, the medial orbitofrontal cortex is related to monitoring the reward value of different (primary and secondary) reinforcers [98]. Within this cluster, the ACC showed valence-specific activation patterns, with enhanced activation for positive compared to negative words. In fact, it has been claimed that electrophysiological signals in the ACC as measured in single neurons (e.g. [99]) “encode motivational aspects of events along a good-bad (and perhaps a better-worse) continuum” ([100], p. 1623). Amongst other functions, the ACC represents the neural basis for the interaction of emotion and attention (for a review, see [101]). In contrast to the VAC, which clearly serves the orienting of attention necessary for processing information, the ACC has been attributed to a general alert state [91]. Thus, overall, our data – in line with our example – suggest that positive words, elicit an alert state only “after” the engagement of directed attention in the VAC and a preliminary lexical analysis initiated during the P1 window.

### Regions Displaying More Activity to Negative Compared to Positive Words

Negative words – compared to equally arousing positive words – elicited enhanced activity in the P1 time window only. They recruited a prefrontal network peaking in the left superior medial frontal lobe and extending to the right ACC (BA 32) and the left middle frontal lobe. The coactivation of these two structures has often been reported in the literature, and has been attributed to wide-spread cortico-cortical connections between the medial frontal lobe and the ACC (for review, see [102]). The apparent paradox is *how* negatively-valenced words can activate this prefrontal network responsible for the encoding of motivational aspects of events [100] and for a general alert response [91] at a point in time when lexical analysis has only just started. This paradox may be solved by assuming emotional tags for word forms that, depending on their valence, *directly* activate these survival relevant structures. There is evidence for a very rapid (<100 ms) modality-independent involvement of prefrontal brain regions in response to negatively conditioned stimuli (for review see, [57]). With MultiCS-conditioning, it was shown that prefrontal regions respond in a highly stimulus-resolving manner and thus allow for a rapid differentiation of learned CS-US from CS-noUS associations. Thus, the prefrontal network establishes a perfect basis for the “emotional tagging” of word forms during word acquisition – as illustrated in our example. Convergent evidence for the temporal sequence of effects in the ACC (i.e. first negative, then positive) comes from Carretié et al. [13] who used an odd-ball paradigm with emotional pictures to study automatic attention as indexed by enhanced ACC activity. In this study, in the P1 (105 ms), only negative pictures received enhanced activation in the ACC whereas in the P2, both positive and negative pictures recruited this structure more than neutral ones. In line with our findings, this study has demonstrated that the spatiotemporal activation of the ACC is modulated by the valence of a stimulus, with a faster time course for negative than for positive stimuli. Note that we observed a stronger ACC activation in response to positive compared to negative words, whereas [13] did not find differences between these conditions during the P2. This might be due to differences in the experimental procedures (RSVP vs. oddball paradigm), or to differences in the material (words vs. pictures).

Why should the activation of this prefrontal “alert system” rely on a lexical analysis in positive, but not in negative words? From an evolutionary perspective, the consequences of reacting slowly to negative (e.g. unsafe or harmful) stimuli are often much more dramatic than the consequences of a similar reaction to positive stimuli [103], [104]. Thus, it makes sense that the prefrontal “alert system” is directly activated by negative events – in our case words – despite the lack of a full lexical analysis of the (negatively tagged) word form.

Still, it remains unclear, what happens *after* this first activation of the prefrontal system. Why is the enhanced activity for negative words only visible in the P1? There are two lines of argument for this finding. First, “a general-purpose defense mechanism that reacts to threat (such as that conveyed by emotional words) by temporarily disrupting all ongoing activity” ([105], p.232) might be active. In this line of argument, one might assume that the linguistic analysis and mechanisms of directed attention in the VAC to negative stimuli are less pronounced, *because* they are suppressed by prefrontal structures being recruited during the P1. However, the findings of our companion study [19] showed that positive and negative words were better recalled than neutral ones, and were associated with enhanced activity in parietal and occipital areas, and in posterior limbic structures. Given this pattern, we consider this explanation rather unlikely. Second, it is possible that the activation of the prefrontal alert system is *sufficient* to secure adaptive responding to negative stimuli, as formulated in the response relevance hypothesis (cf. [38]). In the current study, we cannot distinguish these two alternatives, because our participants were not involved in any tasks during the experiment. Future research employing the same word categories in combination with different tasks may shed light on the functional contribution of these different cortical and limbic structures. A case in point is a behavioral study by Estes and Verges [38], who found a lexical advantage for positive over negative words, as indexed by shorter reaction times in a lexical-decision task. In contrast, in a valence-judgment task, participants were faster to categorize a word as negative [38]. Consequently, the authors have argued that a selective response to negative stimuli can be only found in tasks where stimulus valence is response-relevant.

### Hypothesis of Hemispheric Asymmetry

A further goal of our study was to investigate the hypothesis of valence-specific prefrontal lateralization effects ([47], [48] for a critical review see [49]). In the P1 and P2 interval, we found no evidence for such a lateralization. Valence-specific lateralization of the PFC was in fact found in the later EPN time interval; however, this effect did not survive the Alphasim correction. In the EPN (200–300 ms), we observed more activity for positive than for negative words in the left prefrontal cortex (BA 4, 6, 8, 9, 10) and the reverse pattern, more activity for negative compared to positive words, in the right prefrontal cortex (BA 6, 8, 9). These findings closely resemble an fMRI study that localized asymmetries of positive compared to negative words to the dorsolateral PFC (BA 6, 9) [56] and provide support for the hypothesis of hemispheric asymmetry (e.g., [47], [48]), which links approach-related emotions to left and avoidance-related emotions to right frontal regions. But again, these effects were significant by trend only and should thus be interpreted with caution.

### Conclusions

Taken together, this study shows that the hedonic valence of words is identified early on in the processing stream. Positive words initially predominantly activate language-related structures such as left middle temporal and inferior frontal regions, and the visual association cortex, that is, occipital and parietal regions subserving directed attention processes (cf. [91]). This is then followed by enhanced prefrontal and ACC activity in the P2. By contrast, negative words immediately activate the ACC, which has been attributed to a general alert state (cf. [91]). We have accounted for these effects is in terms of an “emotional tagging” of word forms during language acquisition, which is then followed by different processing strategies, including enhanced lexical processing of positive words and a very fast language-independent alert response to negative words. The valence-specific recruitment of different networks might underlie fast adaptive responses to both approach- and withdrawal-related stimuli, be they acquired or biological.

## Supporting Information

Tables S1 **Includes table S1 and table S2.** Table S1. Description of the stimulus materials used in the experiment. Table S2. Regions of significant activation in the contrast emotional>neutral in the P1 (80–120) and in the EPN (200–300) interval.
(PDF)
