# Supplementary material for: Early Prefrontal Brain Responses to the Hedonic Quality of Emotional Words – A Simultaneous EEG and MEG Study
Source: PLoS One. 2013 Aug 5;8(8):e70788. doi: 10.1371/journal.pone.0070788 (PMC3733636; doi:10.1371/journal.pone.0070788)
Supplement: Tables S1 — Includes table S1 and table S2. Table S1. Description of the stimulus materials used in the experiment. Table S2. Regions of significant activation in the contrast emotional>neutral in the P1 (80–120) and in the EPN (200–300) interval. (PDF) [file pone.0070788.s001.pdf]

How 'Love' and 'Hate' Differ From 'Sleep': Using Combined Electro/Magnetoencephalographic Data to Reveal the Sources of Early Cortical Responses to Emotional Words

Kati Keuper,[1,5]* Peter Zwanzger,[2] Marisa Nordt,[1] Annuschka Eden,[1] Inga Laeger,[2] Pienie Zwitserlood,[3,5] Johanna Kissler,[4] Markus Junghöfer,[1,5] and Christian Dobel[1,5]

[1]*Institute for Biomagnetism and Biosignalanalysis, University of Muenster, D-48149 Muenster, Germany*
[2]*Department of Psychiatry, University of Muenster, D-48149 Muenster, Germany*
[3]*Institute of Psychology, University of Muenster, D-48149 Muenster, Germany*
[4]*Department of Psychology, University of Bielefeld, D-33501 Bielefeld, Germany*
[5]*Otto Creutzfeldt Center for Cognitive and Behavioral Neuroscience, University of Muenster, D-48149 Muenster, Germany*

Abstract: Emotional words—as symbols for biologically relevant concepts—are preferentially processed in brain regions including the visual cortex, frontal and parietal regions, and a corticolimbic circuit including the amygdala. Some of the brain structures found in functional magnetic resonance imaging are not readily apparent in electro- and magnetoencephalographic (EEG; MEG) measures. By means of a combined EEG/MEG source localization procedure to fully exploit the available information, we sought to reduce these discrepancies and gain a better understanding of spatiotemporal brain dynamics underlying emotional-word processing. Eighteen participants read high-arousing positive and negative, and low-arousing neutral nouns, while EEG and MEG were recorded simultaneously. Combined current-density reconstructions (L2-minimum norm least squares) for two early emotion-sensitive time intervals, the P1 (80–120 ms) and the early posterior negativity (EPN, 200–300 ms), were computed using realistic individual head models with a cortical constraint. The P1 time window uncovered an emotion effect peaking in the left middle temporal gyrus. In the EPN time window, processing of emotional words was associated with enhanced activity encompassing parietal and occipital areas, and posterior limbic structures. We suggest that lexical access, being underway within 100 ms, is speeded and/or favored for emotional words, possibly on the basis of an "emotional tagging" of the word form during acquisition. This gives rise to their differential processing in the EPN time window. The EPN, as an index of natural selective attention, appears to reflect an elaborate interplay of distributed structures, related to cognitive functions, such as memory, attention, and evaluation of emotional stimuli. *Hum Brain Mapp 00:000–000, 2012.* © 2012 Wiley-Periodicals, Inc.

Key words: EEG; MEG; P1; EPN; emotion; arousal; words; source reconstruction

Contract grant sponsor: IZKF (awarded to C.D. and P.Z.); Contract grant number: Do3/021/10.

*Correspondence to: Kati Keuper, Institute for Biomagnetism and Biosignalanalysis, University of Muenster, Malmedyweg 15, D-48149 Muenster, Germany. E-mail: k.keuper@uni-muenster.de

Received for publication 26 April 2012; Revised 3 October 2012; Accepted 4 October 2012

DOI: 10.1002/hbm.22220

Published online in Wiley Online Library (wileyonlinelibrary.com).

© 2012 Wiley-Periodicals, Inc.

INTRODUCTION

To ensure survival, evolutionary significant stimuli must be detected rapidly and reliably. Such prioritized sensory processes might be facilitated when motivationally relevant objects or events in our environment engage attention [Vuilleumier, 2005; Vuilleumier and Huang, 2009]. Support for this comes from emotion-sensitive event related potential (ERP) components such as the P1 and the early posterior negativity (EPN, 120–300 ms), observed in electroencephalographic (EEG) and magnetoencephalographic (MEG) studies [for review, see Olofsson et al., 2008]. The motivational connotation of emotional stimuli is mainly determined by two factors, i.e., their valence (pleasant vs. unpleasant) and their arousal (arousing vs. calm). Very pleasant or unpleasant stimuli are usually also rated as being highly arousing [Bradley and Lang, 1994]. The EPN, an index of "natural selective attention" [Olofsson et al., 2008] is elicited by various types of evolutionary significant arousing stimuli like emotional scenes and faces [e.g., Junghöfer et al., 2001; Schupp et al., 2004a, 2004b]. Interestingly, this component and occasionally also even earlier emotional–neutral differentiations, starting already with the P1, have also been found in response to entirely symbolic stimuli that acquire their (emotional) meaning exclusively by learning, such as gestures [Flaisch et al., 2011] and emotional words [Herbert et al., 2008; Kissler et al., 2007, 2009; Scott et al., 2009; for review, see Kissler et al., 2006].

Early emotion effects in word processing give rise to three important issues that this study addresses. First, when does the emotional connotation of such symbolic stimuli become evident during processing, and which subsequent processing steps are influenced by their emotional value? Second, what are the cerebral generators underlying emotion effects in word processing at different critical time points? Third, what is their likely functional significance and what psycholinguistic processes are reflected by these activities?

For emotional words, some studies find initial effects starting with the EPN (>200 ms), others observe an even earlier impact. Kissler et al. [2007], for example, repeatedly presented high-arousing positive and negative nouns and low-arousing neutral nouns in a passive viewing task while recording ERPs. The initial differentiation between emotional and neutral words, found in the EPN (200–300 ms), was interpreted to arise postlexically on the basis of semantic processes. In line with this interpretation, some authors found a morphologically similar negativity to emotional compared with neutral words for even later time intervals, between 300 and 400 ms after word onset [Palazova et al., 2011; Schacht and Sommer, 2009a, 2009b).

However, emotion effects for words have been found as early as the P1 time range (around 80–120 ms), albeit less consistently [e.g., Hofmann et al., 2009; Kissler et al., 2009; Ortigue et al., 2004; Scott et al., 2009; for review, see Kissler et al., 2006]. These early effects are particularly puzzling because they raise an apparent paradox of semantic effects: How can—at a point in time when access to word forms has only just started—emotional valence and arousal be part of a word's meaning? It is unclear whether such very early emotion effects reflect conditioned associations between word forms and emotional connotation [Montoya et al., 1996; Mouchetant-Rostaing et al., 2000; Ortigue et al., 2004; Palazova et al., 2011], or facilitated lexical or lexicosemantic processes [e.g., Hofmann et al., 2009; Scott et al., 2009]. The latter view implies that a word's emotional value is represented lexically, either as part of its semantics or as a lexical variable. The view that a word's emotionality is represented lexically and that this lexical representation can affect very early processing stages is supported by data from Scott et al. [2009], Palazova et al. [2011], and Hofmann et al. [2009]. These authors demonstrate emotion by word-frequency interactions, indicative of lexical effects, already in the P1 time range and extending to the EPN [Scott et al., 2009]. Furthermore, Hofmann et al. [2009], by means of a sLORETA source reconstruction of EEG data, identified left occipitotemporal areas (including the middle temporal gyrus (MTG) and fusiform gyrus) to be involved in P1 emotion effects of negative words in a lexical decision task. The MTG has previously been linked to lexicosemantic processing [for a review see Price, 2000]. Thus, its activation in the P1 time window might reflect speeded lexical access and semantic activation of emotional words. On the other hand, Ortigue et al. [2004] reported bilateral occipital sources of a P1 emotionality effect, with a right-hemisphere current-density maximum for emotional words presented in the right visual field, and a left-hemisphere maximum for all other conditions. Based on this topography, Ortigue et al. [2004] suggested this effect to reflect "rapid activation of neural representations—mnemonic templates—for emotional-word stimuli rather than prelexical semantic processes" (p. 1242). This view is more in line with the assumption that early emotion effects in words reflect conditioned associations [Montoya et al., 1996; Mouchetant-Rostaing et al., 2000; Ortigue et al., 2004; Palazova et al., 2011]. Note that, together, the above studies challenge Kissler et al.'s [2007] view that emotional processing of words emerges only with the EPN (200–300 ms) and that its functional locus is postlexical.

A further crucial question concerns the brain regions that generate the above described scalp-potential fluctuations measured over extended visual cortex. One prominent model for enhanced processing in visual areas assumes 're-entrant processing': during the processing of emotional stimuli, the amygdala is supposed to drive the amplification of perceptual processes by means of reciprocal feedback connections with the extended visual cortex. This model is supported by several functional magnetic resonance imaging studies [fMRI; Anderson and Phelps, 2001; Bradley et al., 2003; Morris et al., 1998, 1999; Sabatinelli et al., 2005; for words see Herbert et al., 2009; Tabert et al., 2001]. By now, it is well established that the amygdala may

respond to arousing negative and/or positive words [e.g., Herbert et al., 2008; Isenberg et al., 1999; Laeger et al., 2012; Straube et al., 2011; Tabert et al., 2001]. Re-entry mechanisms driving visual processing of emotionally relevant stimuli have also been shown for other (cortical) regions such as the parietal cortex [Keil et al., 2009]. Moreover, the posterior cingulate cortex (PCC, part of the limbic cortex) is consistently activated by emotionally salient words [for review, see Maddock, 1999]. This region may support the evaluation of emotional stimuli [e.g., Cato et al., 2004], and is further involved in emotional memory retrieval, and in interactions of emotion and memory processes [Kuchinke et al., 2005; Maddock et al., 2003; for review, see Maddock, 1999]. Finally, frontal brain areas are involved in the emotional evaluation [Dolcos et al., 2004] and in valence processing [Kensinger and Corkin, 2004, Lewis et al., 2007] of emotional words. In sum, electrophysiological and functional-imaging studies reveal enhanced processing of linguistic emotional information in the frontal, the parietal, and the extrastriate visual cortex, as well as in a corticolimbic circuit including the amygdala, and PCC.

However, it is still unclear how the activity of different brain areas maps onto different temporal stages of emotional-word processing. All neurophysiological studies of emotional-word processing with high temporal resolution so far used EEG, and only few studies performed source analysis [Hofmann et al., 2009; Kissler et al., 2007; Ortigue et al., 2004] to reveal underlying neuronal generators. Despite the fact that MEG represents, within one single method, probably the best combination of spatial and temporal resolution of all noninvasive methods in common use [for review, see Hämäläinen et al., 1993; see also Salmelin and Baillet, 2009], to our knowledge there is not a single MEG study of emotional-word processing. Moreover, electromagnetic source localization accuracy can be further improved, and existing discrepancies in the literature potentially reduced, by acquiring EEG and MEG data simultaneously in the same subject and by using realistic head models and combined source reconstruction.[1]

Important factors to improve the source localization of real-time neurophysiological data as provided by EEG and MEG are (1) the consideration of the differential sensitivities of EEG and MEG to different types of cortical signal and (2) the choice of an appropriate head model. First, theoretical considerations show that MEG and EEG are selectively sensitive to particular neural generators. Radial electrical currents with orientations perpendicular to the scalp for instance are poorly detected by MEG but properly represented in EEG measurements. MEG, on the other hand, is especially suitable for measuring magnetic fields generated by tangentially oriented currents [Zschocke, 2009]. Thus, to obtain the full neurophysiological signal, in the current study EEG and MEG were recorded and analyzed simultaneously. Additionally, this combination yields a better localization accuracy than can be afforded by only one type of signal [Goldenholz et al., 2009; Sharon et al., 2007], especially for limbic structures such as the cingulate cortex [Molins et al., 2008]. This improved accuracy is due to both superior spatial sampling and to the complementary properties of EEG and MEG regarding field patterns and sensitivity [Cohen and Cuffin, 1983]. Second, the spherical head model, most commonly used for source localization, might not be appropriate to localize the processes we are interested in, as the resulting accuracy of the source localization is limited, especially in the temporal [Fuchs et al., 2002] and prefrontal cortex [Junghöfer et al., 2006]. This might be due to strong conductivity inhomogeneities and anisotropies [Wolters et al., 2006] and due to extreme deviations in these regions between the realistic head shape and the sphere model. Realistic head models (e.g., boundary element models) derived from the participants' individual anatomies are perfectly suited to overcome these limitations.

Taken together, the current study investigates the spatiotemporal brain dynamics underlying emotional and cognitive processes during word processing by means of a combined EEG/MEG study. This should shed light on (a) the current discussion regarding the distinction of lexicosemantic and conditioned effects (e.g., "mnemonic templates") and (b) the mechanisms of natural selective attention in response to words. Participants read high-arousing positive and negative nouns, and low-arousing neutral nouns, presented without any additional task instruction. We tested for early emotion effects in the P1 time window, where the combination of EEG and MEG data may reveal higher responses to emotional words, although such effects have previously been reported only in more complex designs. Occipital sources would support a nonlexical interpretation of such effects [Ortigue et al., 2004; Palazova et al., 2011], which is in line with an associative conditioning interpretation [Montoya et al., 1996; Mouchetant-Rostaing et al., 2000]. However, if P1 effects were visible in left temporal regions [Hofmann et al., 2009], that is, in structures that underlie lexicosemantic processes [e.g., Lau et al., 2008; Price, 2000], this would support early linguistic processes such as lexical and semantic access [e.g., Pulvermüller et al., 2009; Sereno and Rayner, 2003]. Regarding the EPN time window (200–300 ms), we hypothesized a relative negativity for emotional words at posterior electrodes in the EEG replicating previous studies [e.g., Herbert et al., 2008; Kissler et al., 2007, 2009; Scott et al., 2009]. Its underlying generators were expected to involve areas related to visual attention, and to semantic processing. Finally, to confirm enhanced processing of emotional words at a behavioral level [e.g., Kissler et al., 2007], we predicted higher performance in a free-recall test of emotional compared with neutral words.

[1]It should be noted that many discrepancies of effects found between electrophysiological and hemodynamic measures are direct consequences of their intrinsically different temporal and spatial resolution. Here, we focus on discrepancies which can be reduced by enhancing the coverage of the electrophysiological signal space by EEG/MEG combination.

MATERIALS AND METHODS

Subjects

Twenty right-handed healthy native German speakers (11 female, 9 male; aged 21–31 years) were financially compensated for their participation in this study (nine Euros per hour). All participants were registered in the participants' pool of the Institute for Biomagnetism and Biosignalanalysis (IBB) of the University of Münster, Germany, and were thus familiar with the MEG and EEG testing procedure. All participants gave written informed consent to participate in the experiment, and had normal or corrected to normal vision. Two participants were excluded from the analysis due to strong and continuous artifacts in the EEG-recordings. Thus, data of 18 subjects entered the analysis. The study was approved by the Ethics Committee of the Ärztekammer Westfalen-Lippe and the Medical Faculty of the University of Münster.

Stimuli

Stimuli were taken from the study of Kissler et al. [2007] and consisted of 180 German nouns. Based on emotional arousal and hedonic valence ratings from 45 participants using the Self-Assessment Manikin [Bradley and Lang, 1994], Kissler et al. selected 60 high-arousing positive (*pos*), 60 high-arousing negative (*neg*) and 60 low-arousing neutral (*neut*) nouns. Arousal levels of words with a positive or negative valence did not deviate significantly. All three word categories were matched for length, frequency of use [based on the CELEX database; Baayen et al., 1995], concreteness, and neighborhood size (obtained from the dlex
T1 database under http://dlexdb.de/; see Table I).

Procedures

The experiment was carried out at IBB at the University of Münster. All participants received written instructions on the experimental procedure. The individual EEG-electrode positions for each subject were digitized by means of a 3D tracking device (Polhemus, Colchester). Participants were then seated in the sound-attenuated, dimly lit, and magnetically shielded MEG chamber. Three landmark coils (two auditory channels and the nasion) were digitized using the Polhemus 3Space® Fasttrak to determine the head position in the MEG scanner. Head motion within the simultaneous MEG/EEG acquisition was below 0.5 cm for all participants.[2] Participants were instructed to carefully read the words that were presented visually in black characters on a light gray screen by means of the software Neurobehavioral Systems Presentation® (www.neurobs.com).

[2]Head motion was determined by measuring the maximal Euclidian distance between fiducial positions when the run began and their positions throughout the run in the individual headframe coordinate system.

TABLE I. Description of the stimulus materials used in the experiment [taken from Kissler et al. 2007]

	Word category					
	Pleasant		Neutral		Unpleasant	
Variable	*M*	SD	*M*	SD	*M*	SD
Valence	7.40	0.12	5.10	0.05	1.90	0.09
Arousal	5.70	0.12	2.20	0.10	5.80	0.11
Concreteness	4.51	0.17	4.02	0.26	4.09	0.18
Word length (letters)	7.70	0.37	7.40	0.35	7.30	0.32
Word frequency (per million)	97.48	17.91	108.78	15.83	60.01	22.67
Neighborhood size (orthographic)	10.65	15.30	9.22	12.12	8.80	13.52

M, mean, SD, standard deviation.

Viewing distance was 90 cm, with an average visual angle of 3.93° (center to edge, SD = 1.24°). Each participant was shown five repetitions of differently randomized rapid serial visual presentation (RSVP) sequences, consisting of 180 words each. Thus, overall 300 words per emotional category were presented. Transitional probabilities between the three emotional conditions (positive, negative, neutral words) were balanced within each sequence. Additionally, sequence order was counterbalanced across subjects. Each word was presented for 1,000 ms (1 Hz), followed immediately by the next item. This RSVP procedure allowed the presentation of multiple stimuli within a relatively short experimental session, lasting for only 15 min. The resulting high signal-to-noise ratio is particularly important for a precise source reconstruction. Due to the rapid succession of stimuli, brain responses to subsequent trials might overlap. However, with complete randomization potential overlaps from preceding trials are identical in all affective conditions. Based on self reports, subjects had no difficulty in reading the word stimuli. Immediately after the measurement, participants were given an unannounced free-recall task in which they had to write down as many of the presented words as they could remember without time restriction.

MRI Protocol

To construct individual boundary-element models for the current density reconstruction performed on the EEG-MEG-Data, we recorded T1-weighted anatomical scans for each participant in a 3-Tesla Scanner (Gyroscan Intera T30, Philips, Amsterdam, Netherlands). Using Turbo Field Echo acquisition, 400 contiguous T1-weighted (TR=7.33.64 ms, TE=3.31 ms) 0.5-mm-thick slices in the sagittal plane were obtained. The field of view was set to 300 mm × 300 mm, with an in-plane matrix of 512 × 512. Therefore, the native voxel size was 0.5 mm × 0.58 mm × 0.58 mm.

Preprocessing of these images included an intensity bias regularization, to account for differences in the intensity of MR-images within each type of tissue, and an additional spatial reslicing to isotropic voxels (1.17 mm × 1.17 mm × 1.17 mm). Both steps were performed by means of the SPM8 software package (Statistical Parametric Mapping, http://www.fil.ion.ucl.ac.uk).

EEG and MEG Recordings and Parametrization

MEG signals were recorded continuously by means of a 275-sensor whole-head MEG-system (Omega 275, CTF, VSM MedTech) with first-order axial SQUID gradiometers (2 cm diameter, 5 cm baseline, 2.2 cm average inter-sensor spacing). Simultaneously, EEG was recorded continuously from 81 channels using a CTF Omega system (VSM MedTech, Coquitlam, Canada). 80 electrodes, including six ocular electrodes, were mounted on a flexible lycra-electrocap (easycap, Falk Minow Services, Munich Germany), placed in accordance with the extended version of the international 10–10 system, and referenced to Cz during recording. In addition, electrocardiogram (ECG) electrodes were attached to the subjects' right cervix and left costal arch. Electrode impedances were kept below 8 kΩ. MEG, EEG and ECG signals were digitized with 600 Hz sampling frequency and on-line low-pass filtered at 150 Hz.

Preprocessing of EEG-MEG-Data

Recordings were further processed offline with Brain Electrical Source Analysis 5.3 software. Data were filtered using a 40 Hz low-pass and a 1 Hz high-pass filter. As discussed in Peyk et al. [2008], high-pass filters allow a baseline-independent investigation of short-latency ERFs, because they attenuate effects of residual processing from the preceding trial, and highlight short-term and phasic changes of neural activity. As such, the high-pass filter was taken as an alternative to the standard baseline correction. EEG and MEG correlates of ocular activity were corrected by applying the adaptive artifact-correction method recommended by Ille et al. [2002]. EEG electrodes with sustained artifact contamination were interpolated, if fewer than six sensors not adjacent to each other were affected. Otherwise, the participant was excluded from further analyses. The averaging epoch for each trial lasted from 200 ms before stimulus onset to 800 ms after stimulus onset. To avoid phasic artifact contamination, trials exceeding a magnetic field strength of 3,000 pT in the MEG, or a potential of 120 μV in the EEG, at any sensor were excluded from the subsequent analysis. The number of excluded trials did not differ across the emotional-word conditions ($F(2,34) = 1.42$, $P = .26$; .26 (Greenhouse-Geisser corrected)). Averages were computed separately for each condition, and subsequently down-sampled to 250 Hz. As in Kissler et al. [2007], the averaged time courses for different emotional-word categories at posterior EEG-electrodes served to define critical time intervals. To replicate previous ERP findings, we computed a repeated measures analysis of variance (ANOVA) with a priori contrasts on the evoked potentials in nine left lateralized posterior electrodes (O1, O9, PO3, PO07, PO9, P3, P5, P7, and P9) and the corresponding right hemispheric electrodes (O2, O10, PO4, PO8, PO10, P4, P6, P8, and P10) for both the P1 and the EPN time interval, using the Matlab-based (The MathWorks) EMEGS software [Peyk et al., 2011; freely available at http://www.emegs.org/]. This ANOVA contained the factors *emotion* (negative vs. positive vs. neutral) and *hemisphere* (left posterior vs. right posterior electrodes).

Current Density Reconstruction

Current source density reconstructions of the underlying neural generators were carried out using the CURRY software package (version 6; Compumedics Germany GmbH, Hamburg). Individual EEG and MEG sensor positions and the subject's structural MRI were coregistered by aligning the anatomical landmarks (nasion, left and right preauricular points). As volume conductor model, a three-compartment boundary element model comprising skin, skull, and brain was computed for each participant. A mesh size of 9, 8, and 6 mm and conductivity values of 0.33, 0.0042, and 0.33 S/m, were chosen for the skin, skull and brain boundary elements, respectively [Geddes and Baker, 1963]. To constrain source space for the inverse solution to the cortex, we modeled a representation of the cortex including the hemispheric fissure on the basis of the gray matter segmentation. Brainstem and cerebellum were excluded. We used a cortical triangle mesh with 3 mm triangle side length, which yielded between 11,410 and 18,591 dipole locations ($M = 14,863$, $SD = 1,911$), depending on the individual brain anatomies. Combined (EEG and MEG) current-density reconstructions (CDRs) were computed using the minimum norm least squares (L2-Norm) method [see Hämäläinen and Ilmoniemi, 1994]. The L2-Minimum Norm Estimate (L2-MNE) is an inverse method that allows the reconstruction of distributed neural sources underlying the event-related magnetic fields and electric potentials, without the necessity of a priori assumptions regarding the number, orientations and possible locations of underlying neural generators. The L2-MNE is calculated by multiplying the pseudoinverse of the so-called lead-field matrix (which describes the sensitivity of each sensor to the sources) with the averaged recorded data. The Tichonov regularization parameter lambda, needed for the calculation of the pseudoinverse, was based on an estimation of an individual noise level within a pre-defined baseline interval ranging from 150 to 50 ms before stimulus onset. To account for different noise levels between EEG and MEG we applied a whitening procedure on the basis of the noise variance estimated from this interval [Fuchs et al., 1998]. Due to the completely randomized stimulus sequence with equal transitional probabilities for each condition, the

interval of 150–50 ms before stimulus onset can be considered an experimentally controlled baseline which does not contain any condition specific expectancy effects. To account for the depth dependency of L2-MNE, a square root compensation was applied [Fuchs et al., 1999]. It was shown that depth weighting can partly compensate for the systematic depth mislocalization of the standard MNE and may also improve the overall localization accuracy [Lin et al., 2006]. However, this comes at the expense of spatial resolution [Lin et al., 2006, for detailed discussion, see Hauk et al., 2011]. Mean L2-norm solutions for the a priori defined P1 (80–120 ms) and EPN (200–300 ms) time intervals of interest were computed separately for each condition and subject. As mentioned above, these time intervals were chosen based on the current literature [e.g., Herbert et al., 2008; Hofmann et al., 2009; Kissler et al., 2007, 2009; Ortigue et al., 2004; Scott et al., 2009], and on the means across subjects plotted separately for each of the three conditions. With calculation of the vector length of the estimated sources, direction independent CDRs were obtained. To eliminate individual differences in brain structure, individual CDRs were normalized to a standard space, using the SPM8 software package [for more details on the normalization procedure, see Bruchmann et al., 2010]. In the next step, CDRs were masked and smoothed with an adapted template of the cerebral cortex restricted to gray matter.

Voxel-wise One-Way ANOVAs (within-subject) were carried out separately for the CDRs of the two time intervals, using the SPM8 software package to test for differential neural generator amplitudes across different experimental conditions.

To correct for multiple comparisons, we used the Alphasim [Cox, 1996] implementation in REST [Song et al., 2011] to determine a spatial cluster-extend threshold. Based on 5,000 Monte Carlo simulations, the probability for a cluster to occur was estimated. For the 8 mm-smoothed (full width at half maximum) images, a cluster was defined as a group of voxels with P-values of ≤ 0.001 that were separated by no more than one voxel width (i.e., 3 mm). For the estimation, we took into account all voxels that were considered as possible sources of the CDRs, by applying the mask used on the CDRs (see above). This procedure yielded a minimum cluster size of 114 voxels for a cluster P-value of ≤ 0.001.

Recall Performance

Performance in the unannounced free recall test was analyzed by means of repeated-measures ANOVA with the factor *emotion (positive vs. negative vs. neutral)*. Separate ANOVAs were conducted for correctly recalled words and erroneous intrusions.

RESULTS

Recall Performance

In free recall, participants correctly reported 28.1 (SD = 15.7) out of the 180 presented words, i.e., 15.6%. Participants correctly recalled 11.9 positive (SD = 5.5), 9.9 negative (SD = 6.2), and 6.2 neutral words (SD = 5.4). A one-way ANOVA (within subjects) on the sums of the correctly recalled words revealed a highly significant main effect for the factor *emotion* ($F(2,34) = 18.5$, $P < .001$). Thus, performance in this unannounced free recall strongly depended on the emotional category of the word, with superior recall for emotional relative to neutral words. As hypothesized a priori, this was true both for positive words ($t(17) = 5.0$; $P < .001$ (one-tailed)) and negative words ($t(17) = 4.1$; $P < .001$ (one-tailed)). *Post hoc* comparisons (Bonferroni corrected) comparing positive and negative words revealed a bias toward positive words, though only marginally significant ($t(17) = 2.1$; $P = .064$ (two tailed)). The ANOVA on the intrusion rate (M = 3.4, SD = 6.1) did not yield any differences between the three emotion categories ($F(2,34) = 0.64$; $P = .53$, .46 (Greenhouse-Geisser corrected)).

ERP and ERF Data in Sensor Space

To compare our results with earlier ERP studies we performed an analysis in sensor space for the EEG data. Figure 1a shows evoked potentials during reading of positive, negative, and neutral words. The graphs display the time course of cortical activation at 18 posterior EEG-electrodes displayed on an average reference montage. In all three conditions, there is clear positive deflection, peaking at 100 ms after stimulus onset, which is most pronounced in occipital sensors (O1, O2, O9, O10). Thus, we set the critical time-window of the P1 component to 80–120 ms. As confirmed by the repeated measures ANOVA with the factors *emotion (positive vs. negative vs. neutral)* and *hemisphere (left posterior vs. right posterior electrodes)* the EEG did not uncover any emotion specific effects in the P1 component (main effect *emotion*: $F(2,34) < 1$; ns, interaction *emotion*hemisphere*: $F(2,34) = 1.02$; ns).

Further, as expected from the current literature [e.g., Herbert et al., 2008; Kissler et al., 2007, 2009; Scott et al., 2009; for review, see Kissler et al., 2006] Figure 1 shows that regardless of the word's hedonic valence, emotional words cause a stronger negativity than neutral words between 200 ms and 300 ms. A repeated measures ANOVA with the factors *emotion (positive vs. negative vs. neutral) and hemisphere (left posterior vs. right posterior electrodes)* in a time interval ranging from 200 to 300 ms revealed a main effect of *emotion* ($F(2,34) = 21.73$, $P < .001$) and an interaction of *emotion*hemisphere* ($F(2, 34) = 5.29$, $P < .001$). As hypothesized a priori, compared with neutral words, both positive ($t(17) = 6.41$, $P < .001$) and negative ($t(17) = 5.40$, $P < .001$) words elicited a stronger negativity. The difference of (positive and negative) emotional and neutral (emo–neut) words was more pronounced in the left compared with the right hemisphere ($t(17) = 5.04$, $P < .001$).

Figure 1b displays the corresponding time course of the ERFs as the global power of left anterior, left posterior, right anterior and right posterior sensors. Note that the

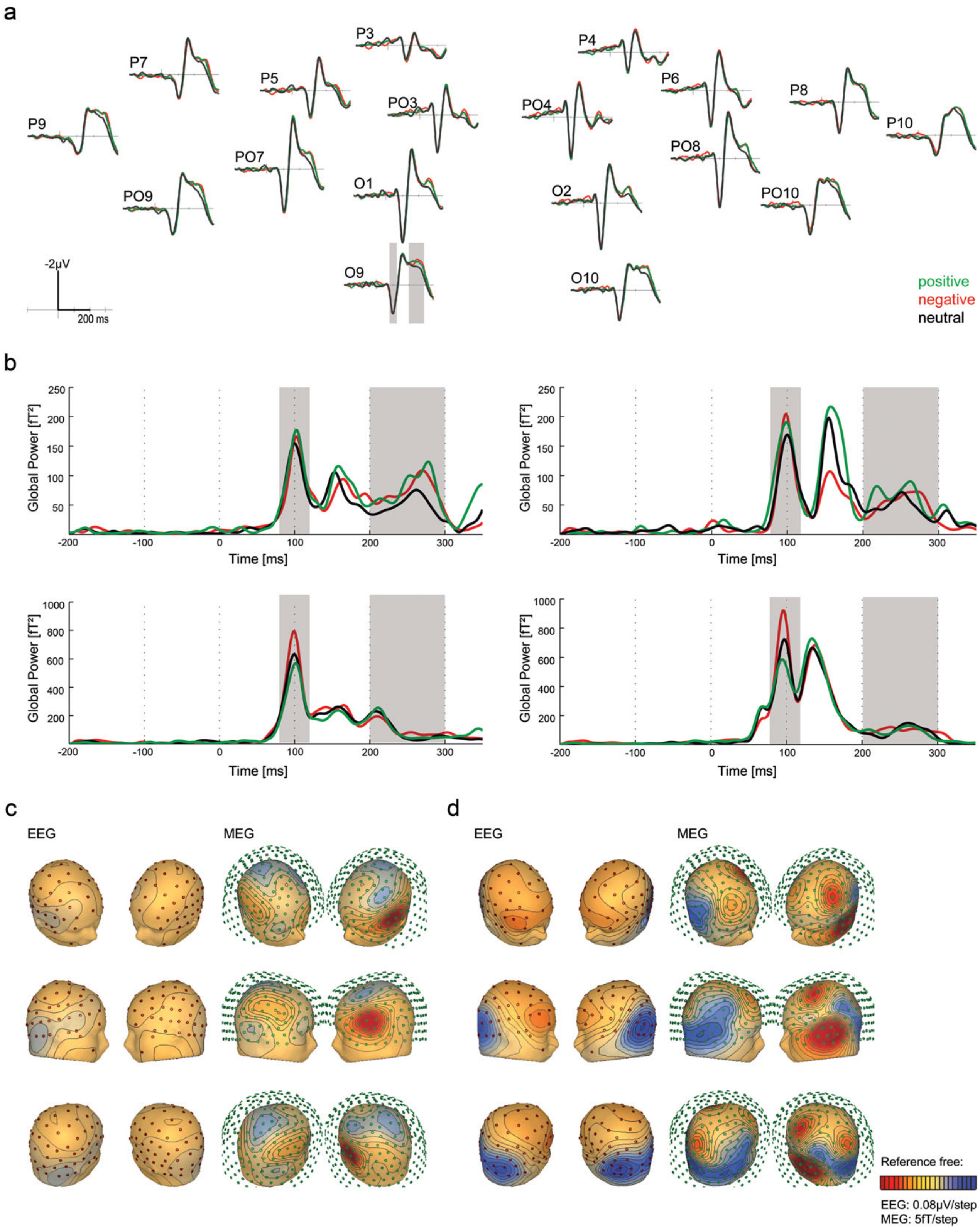
a
P3
P7
P5
P9
PO3
P4
PO4
P6
P8
P10
PO7
O1
PO8
PO9
O2
PO10
O9
O10
-2µV
200 ms
positive
negative
neutral
b
Global Power [fT²]
Time [ms]
c
d
EEG
MEG
Reference free:
EEG: 0.08µV/step
MEG: 5fT/step

Figure 1.

M1, as the MEG correlate of the P1, is also strongly pronounced between 80 and 120 ms in posterior sensors and appears to be strongly modulated by *emotion*. During the time range of the EPN the modulation by *emotion* is not as clearly visible as in the ERPs, but more prominent at anterior compared with posterior sensors.

To display the topography of the *emotion* effect, the positive and negative conditions were collapsed to obtain the average response to emotional words (*emo*). Then, the scalp-potential differences of emotional versus neutral words (*emo–neut*) were computed. Potential and magnetic field topographies of these differences are shown for each EEG and MEG, and separately for the P1 (Fig. 1c) and the EPN (Fig. 1d) time intervals. As confirmed by the ANOVA on ERPs in posterior sensors, there were no observable P1-emotion effects in the EEG. However, the ERFs clearly showed bilateral difference activities that seem to be left-hemispheric dominant, with strongest enhanced outgoing (positive, red) fields over left temporal regions. Figure 1d displays the EEG scalp (left) and MEG field (right) distributions of the difference potentials/fields between emotional and neutral words at its peak in the EPN time window (i.e., 270ms after word onset). The EEG scalp difference is most pronounced at posterior electrodes and slightly lateralized to the left side. The corresponding MEG field-distribution shows multiple and widely distributed areas, with enhanced in- and outgoing difference fields, which suggests that several neural generators are simultaneously active.

Combined EEG-MEG CDR

Statistical analyses of the P1 and the EPN reported in the following were performed on the combined EEG-MEG CDRs for these time windows. All analyses were based on planned contrasts in a one-way ANOVA (within-subject), with the significance level set at $P < .001$ (Alphasim corrected, with a minimal cluster extent threshold of 114 contiguous voxels). Table II shows the clusters with a stronger activation to emotional compared with neutral words. In the P1 time window, we found the left MTG to be more active for emotional than for neutral words. The other contrasts (*neut* > *emo*, *pos* > *neg*, *neg* > *pos*) did not meet our significance criterion. In the time range of the EPN, several clusters showed enhanced activity in response to emotional when compared with neutral words. The statistically most significant local maximum was found in the left supramarginal gyrus ($t = 4.83$; $P < .001$, Alphasim corrected). Other significantly activated regions included the occipital lobe (cuneus and precuneus) and the limbic lobe (PCC). The locations and T-values of peak activation for these activated regions are shown in Table II. Again, in the EPN time window, the other contrasts (*neut* > *emo*, *pos* > *neg*, *neg* > *pos*) did not meet our significance criterion.

For visualization, the uncorrected group-activation maps of the contrast *emo* > *neut* were overlaid on a standard brain from a single normal subject (MRIcroN: ch2.nii.gz) in Figure 2a and b for the P1 and EPN time window, respectively.

DISCUSSION

We set out to investigate the neural dynamics underlying emotional-word processing using a silent reading task. Effects of emotional content were behaviorally assessed by an unannounced free recall test. By using EEG and MEG simultaneously and performing a combined source analysis on the basis of realistic head models with a cortical constraint, we sought to optimize the spatial resolution of the current density reconstruction. We observed enhanced neural activity in response to emotional words during the P1 (80–120 ms) and the EPN (200–300 ms) time windows, irrespective of the word's valence. Combined EEG-MEG source analysis revealed the left MTG to generate the P1 effect, and a distributed network comprising parietal, occipital, and posterior limbic structures to evoke the EPN effect. This enhanced neural activity was accompanied by a better memory for emotional words. In line with Kissler et al. [2007], positive and negative words were better recalled than neutral words in the free-recall test. In the following, we will discuss the P1 and EPN effects in the

Figure 1.
(**a**) Evoked potentials (ERPs) during reading of positive (green), negative (red), and neutral words (gray). The graphs display the time course of cortical activation at 18 posterior EEG-electrodes displayed on an average reference montage. In the electrode O9 the critical time intervals (P1 and EPN) for the CDRs are shaded in gray. (**b**) Evoked magnetic fields (ERFs) during reading of positive (green), negative (red), and neutral words (gray). The graphs display the global power obtained from left anterior (top left), right anterior (top right), left posterior (bottom left), and right posterior (bottom right) sensors. The critical time intervals (P1 and EPN) for the CDR that were selected on the basis of the ERP data are shaded in gray. (**c**) Scalp/field distribution of the scalp/field potential difference (emotional–neutral) at 100 ms (peak of the P1) for EEG (left) and MEG (right), respectively. The red dots indicate EEG-electrodes (left) and the green circles represent MEG sensors (right). Cooler colors indicate more negative-going potentials/fields, whereas warmer colors display more positive-going potentials/fields. (**d**) Scalp/field distribution of the scalp/field potential difference (emotional–neutral) at 270 ms (peak potential difference wave in the EPN time window) for EEG (left) and MEG (right), respectively. The red dots indicate EEG-electrodes (left), the green circles represent MEG sensors (right). Cooler colors indicate more negative-going potentials/fields whereas warmer colors display more positive-going potentials/fields.

TABLE II. Regions of significant activation in the contrast emotional > neutral in the P1 (80–120) and in the EPN (200–300) interval

Effect		Brain region	BA	Cluster size	MNI coordinates of local maximum			
					X	Y	Z	T
P1 (80–120 ms)	L	Middle temporal gyrus	21, 22, 20, 13	143	−58	−20	−15	3.95
EPN (200–300 ms)	L	Supramarginal Gyrus	40	244	−40	−46	35	4.83
	R	Cuneus	19, 18, 7, 31	536	26	−82	15	4.15
		Cuneus	19		12	−94	19	3.71
		Precuneus	19		26	−76	33	3.62
	L	Lingual gyrus	30, 19, corpus callosum, 29, 23, 27, 18, hippocampus, 35, 36, pulvinar, 28, caudate tail, 31	1432	−12	−32	−7	4.10
	B	Posterior cingulate	29		−4	−52	7	3.94

X, *Y*, and *Z*, coordinates according to MNI stereotactic space; BA, approximate Brodmann's area; L, left; B, bilateral; R, right; *T*, peak *T* value; cluster size in voxels ($P < 0.001$ (Alphasim corrected)).

light of the current literature and suggest an integrative account for effects observed in emotional-word processing.

P1. We observed enhanced activation in response to emotional words in the time range of 80 to 120 ms [cf. Hofmann et al., 2009; Kissler et al., 2009; Ortigue et al., 2004; Scott et al., 2009; for review, see Kissler et al., 2006]. So far, P1 effects have been considered more likely in tasks requiring active responses [e.g., Kissler et al., 2009], but we obtained them in a passive reading task. The fact that we could not find evidence for P1 effects in EEG sensor space data replicates Kissler et al. [2007, 2009]. More importantly, it highlights the importance of combined EEG-MEG measures, to fully exploit all available information. Visual inspection of the time course (cf. Fig. 1b) and scalp/field distributions of emotional-word processing at 100 ms (cf. Fig. 1c) suggests that the MEG recordings mainly contributed to our effect. In line with the left-lateralized emotion effect revealed by MEG sensor space data, combined EEG-MEG source estimation localized the P1-effect in the left temporal lobe, with a peak in the left MTG (BA 21).

Which processes might the MTG reflect during the P1 time window? There are two lines of argument for such early effects of emotional words: (a) a separate representational network for stimuli that have acquired emotional status by means of (conditioned) associations between a form (a face, a word form, a pseudoword) and an emotional value [Montoya et al., 1996; Mouchetant-Rostaing et al., 2000; Ortigue et al., 2004; Palazova et al., 2011], or (b) the view that a word's emotional value is represented lexically [e.g., Hofmann et al., 2009; Scott et al., 2009], either as part of its semantics or as a lexical variable, similar to a word's frequency of occurrence, its length, or the density of its lexical environment [e.g., Hauk et al., 2009; Pulvermüller, 2001; Sereno et al., 1998]. Evidence for the first view comes from Ortigue et al. [2004], who found right-lateralized occipital activation for emotional words at about 100 ms. In line with the idea of learned associations, Montoya et al. [1996] found larger left-lateralized effects around 100 ms for pseudowords (phonotactically legal letter strings that have no meaning) that had previously been conditioned with painful electric shock.

What challenges the view of separate neural representations of the (conditioned) association of words and their emotional value are the early effects of lexical information (such as word frequency, length) and, importantly, their interaction with emotional variables in an early time window [e.g., Assadollahi and Pulvermüller, 2003; Hofmann et al., 2009; Scott et al., 2009; Sereno et al., 1998]. In our view, these findings favor the second interpretation, with emotion as a kind of information attached to lexical representations, influencing lexical processes in the MTG as early as 80–120 ms poststimulus onset. In what follows, we present evidence for lexical processing as early as the P1, and for the general functional role of the MTG in lexical processing. Finally, we will try to reconcile the two views presented above.

One way to investigate the onset of lexical processing is to compare existing words and pseudowords. If, other things such as stimulus length being equal, there are differences between words and pseudowords, this indicates that access to lexical representations (lexical access) has taken place. Indeed, such differences between words and pseudowords have been found within 110 ms after word onset [Sereno et al., 1998; see also Hauk et al., 2012; Sereno et al., 2003]. Furthermore, other psycholinguistic variables

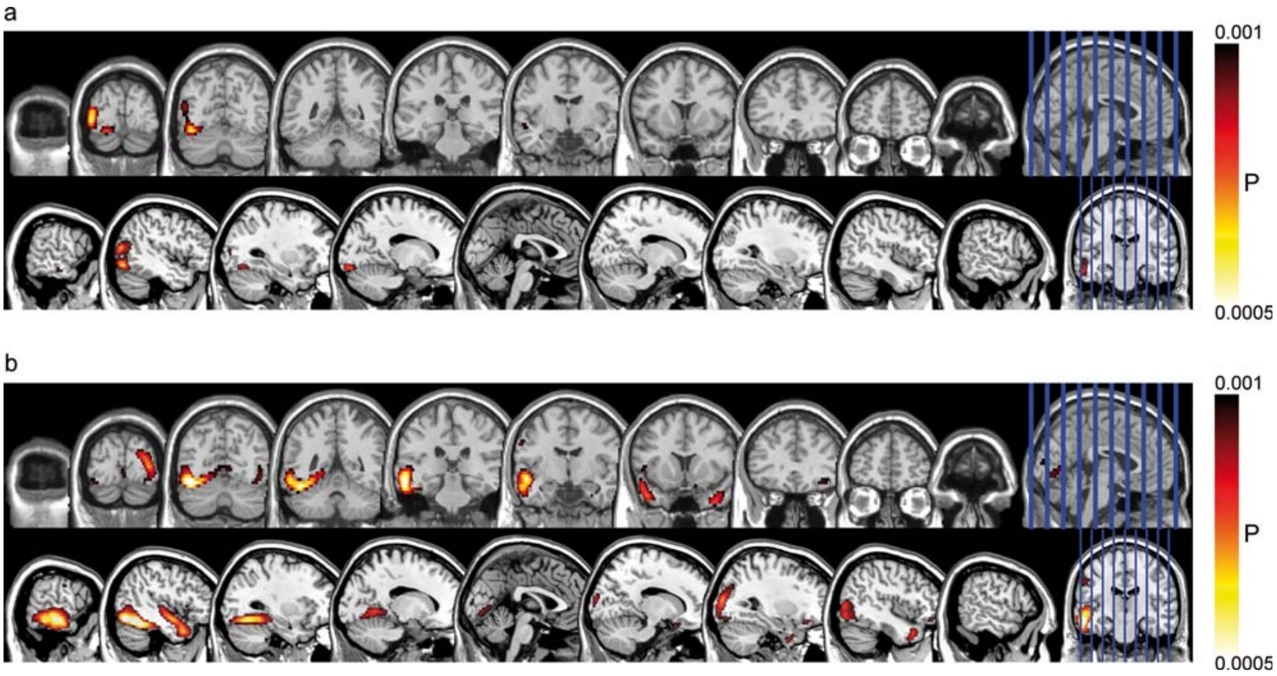

Figure 2.

Cortical regions displaying enhanced activation to emotional compared with neutral words in the P1 (**a**) and the EPN (**b**) time windows. These images were thresholded using a voxel-wise statistical height threshold of ($P < 0.001$). Functional images are superimposed on a standard brain from a single normal subject (MRIcroN: ch2.nii.gz).

(e.g., frequency, spelling regularity, word class, lexical neighborhood density) were found to affect very early ERPs [Hauk et al., 2006, 2009; Sereno et al., 1998; Skrandies, 1998]. Some of these findings were supported by eye-movement data, which underlines their reliability. In a very recent combined EEG-MEG study applying L2 minimum norm source estimates, Hauk et al. [2012] localized lexicality effects (words vs. pseudowords) to the middle left temporal pole, although slightly later than our P1 effect. This study also provided evidence that lexical and semantic information are retrieved almost simultaneously, both supported by brain activity in the left temporal pole.

A lexical interpretation of our P1 effect implies that lexical access is affected by the emotional status of words, either because the emotional value is connected to the word forms themselves, or because emotional connotation, as part of a word's semantic information, becomes available early on, in parallel to word-form information [see Hauk et al., 2012; Sereno and Rayner, 2003; Zwitserlood, 1989; for a review, see Pulvermüller et al., 2009]. A lexical interpretation of our P1 effects fits very nicely with their localization, and with the functional role of the MTG in word processing. According to Lau et al. [2008], the MTG, and the adjacent superior temporal sulcus and inferior temporal cortex, are the regions that serve to store and activate lexical representations. Thus, it is not surprising that lexical and semantic processes, as traditionally indexed by the N400, have often been linked to the left temporal lobe [e.g., Dobel et al., 2010; Hirschfeld et al., 2011; for reviews, see Kutas and Federmeier, 2000; Lau et al., 2008]. In line with this, it has often been suggested that the MTG plays an important role in language comprehension at the word level [for reviews, see Poeppel and Hickok, 2004; Price, 2010]. Additional evidence comes from clinical studies on patients with different language impairments, and from functional imaging studies. Dronkers et al. [2004] demonstrated that patients with lesions in the MTG were most severely impaired in comprehension tests, and thus inferred that their deficit emerged at the level of word comprehension. In addition, two meta-analyses of brain-imaging data revealed the MTG as a core structure for lexical processing, both in language comprehension [Indefrey and Cuttler, 2004] and production [Indefrey and Levelt, 2004]. It thus seems that the MTG houses (parts of) the mental lexicon shared by language comprehension and production. In sum, the localization of our P1 effect of emotional words, the earliness of other effects that cannot be but lexical, tip the scale in favor of a lexical interpretation of our P1 effect.

If our P1 effect is lexical, and all other aspects (word length, frequency, etc.) being equal, how can the lexical system possibly know that "hate" is an emotional item,

whereas "gate" is neutral? A first option is an early, fast emotion-detection mechanism, modulated by the amygdala that allows for rapid preparation of motivational systems [e.g., LeDoux, 2000]. Based on data from rodents, LeDoux suggested that affect-related information can be conveyed to the amygdala via direct projections from the sensory thalamus, with response latencies around 20–30 ms [LeDoux, 2000]. The amygdala shares numerous reciprocal connections with many information-processing areas, including the entire ventral visual stream [Freese and Amaral, 2005; Ghashghaei and Barbas, 2002] that could have subserved the P1 effect in our study. However, Pessoa and Adolphs [2010] challenged the model of a fast thalamoamygdala pathway in humans: intracranial recordings of the human amygdala uncovered the earliest single-unit-responses to visual stimuli around 200 ms [Mormann et al., 2008; Oya et al., 2002]. At about the same time, the amygdala activity was found to be modulated by the emotional content of stimuli [Krolak-Salmon et al., 2004]. Even in the monkey amygdala, evoked differences in response to emotional vs. neutral faces were not found before 120 ms [Gothard et al., 2007]. In line with this, the estimated generators for underlying the P1 (<120 ms) effect did not show any evidence for a limbic involvement—in contrast to the EPN (200–300 ms). Thus, a subcortical pathway is unlikely to produce, at least in an online manner, the early effects seen in this study.

A second way to think of how lexical processing is influenced by emotional connotations is to integrate the ideas put forward by Ortigue et al. [2004]. Due to the concurrent exposure to a word form and its emotional meaning/an emotional state, a more permanent connection between form and emotional connotation may be established, in the sense of an "emotional tagging" [Damasio, 1996] of the word form—most probably supported by natural selective attention [Olofsson et al., 2008] and by limbic structures [Bechara et al., 1995]. This "tagging" becomes neurally represented, as "mnemonic templates" [Ortigue et al., 2004], or as a conditioned association between a word's form and the emotional connotation [Palazova et al., 2011]. The emotional tag is immediately available on presentation of the word form. In this view, emotionality could be considered a lexical variable, like word frequency or word length. Independent of its exact neural source, its effects would be visible in word-processing areas (MTG), as soon as lexical access is initiated. Similarly to high-frequency words, lexical access and semantic analysis could then be facilitated and/or speeded for emotionally tagged words reflected by an enhanced EPN. The output of this lexicosemantic analysis could then give rise to processes of (natural) selective attention and prioritized processing of emotional words, as reflected in the EPN.

Recently, Steinberg et al. [2012]—by applying a novel MultiCS conditioning paradigm—revealed a similar "emotional tagging" of different faces in an early interval even preceding the P1 [for review, see Steinberg et al. in press]. In this context, the related question arose how emotional face categorization could precede processing stages necessary for face identification. The authors argued that this early response reflects a fundamental discrimination of "emotional" from "neutral" or "relevant" from "irrelevant" [Lang et al., 1997], which would then trigger preferential processing of emotional faces in the EPN time interval. The rapid affective face categorization of more than 100 faces after only two learning instances in this study points to highly resolving visual processing in this early interval. Such a high resolution would explain an easy emotional discrimination of words like "hate" and "gate" and lends support to the thesis that the perceptual system rapidly differentiates even perceptually very homogeneous objects according to their emotional significance. It also implies that an emotional discrimination of newly learned words can be acquired after only few learning instances.

EPN. Replicating Kissler et al. [2007], we found enhanced activation in response to emotional words in the time range of 200–300 ms after stimulus onset (EPN) at posterior EEG electrodes. The EPN reflects enhanced perceptual processes to emotional stimuli [e.g., Flaisch et al., 2011; Junghöfer et al., 2001; Kissler et al., 2007; Schupp et al., 2004a, 2004b), probably on the basis of (natural) selective attention mechanisms [e.g., Olofsson et al., 2008]. As discussed above, this amplification of perceptual processes most likely reflects reciprocal feedback processes driven by the amygdala [e.g., Anderson and Phelps, 2001; Herbert et al., 2008; Sabatinelli et al., 2005] or other upstream structures. In the current study the statistical analysis of the CDRs revealed a more detailed view on the generators of this component in word processing: the EPN effect was triggered predominantly by three brain structures. First, in line with the current literature, we found occipital regions, comprising secondary and associative visual areas, to be more activated by emotional words. Second, the left supramarginal gyrus (BA 40) (located in the inferior parietal lobe) strongly differentiated between emotional and neutral words. BA 40 is activated by the emotional salience of words [Kensinger and Corkin, 2004], working memory processing of emotional content [Rämä et al., 2001], and visual attention [for review, see Culham and Kanwisher, 2001]. Most interestingly, the third cluster peaked in posterior limbic structures and extended to occipital, temporal and sublobar areas. Within this cluster, the strongest difference between emotional and neutral nouns was located in the PCC, particularly the retrosplenial cortex, comprising BA 29 and BA 30 [Maddock, 1999]. The PCC is a rather deep region that has previously been linked to the evaluation of external emotional stimuli, including words [e.g., Cato et al., 2004], to emotional memory retrieval, and to interactions of emotion and memory processes [Kuchinke et al., 2005; Maddock et al., 2003; for review, see Maddock, 1999]. Thus, the present pattern of results is well in line with the concept of "(natural) selective attention" and with the neuroimaging literature on emotional-word processing. Besides the good fit with the fMRI literature, there are several methodological

arguments that indirectly support the localizatory and statistical validity of our results. First, there are some studies that demonstrated the MEG's ability to detect activity from deep structures [Ioannides et al., 1995; Luo et al., 2007, 2009; Streit, 2003]. Second, particularly for the cingulate gyrus, Molins et al. [2008] reported an improved resolution of the L2-MNE on the basis of simultaneous EEG and MEG as compared with MEG alone. Third, data were analyzed in a very conservative manner: for CDR, activation within an extended time window of 100 ms was averaged and corrected for multiple comparisons.[3]

Taken together, we suggest that lexical access is speeded for emotional words, possibly on the basis of an "emotional tagging" of the word form during earlier exposure and acquisition. This facilitation of lexical processes starts as early as 80–120 ms (P1) and is reflected in enhanced activity for emotional words compared with neutral words over the left MTG. This initial prioritized processing for emotional words is followed by a later, similar effect encompassing parietal, visual, and posterior limbic structures (EPN, 200–300 ms). We suggest that the EPN, as an index of "natural selective attention" [Olofsson et al., 2008] reflects an elaborate interplay of distributed neocortical and limbic structures, related to cognitive functions such as memory, attention and evaluation of emotional stimuli. Such a biphasic stage of processing has been recently also evidenced for in other domains of emotional processing, e.g., processing of aversively conditioned faces [Steinberg et al., 2012; for a recent review see Steinberg et al. in press). Thus, it is likely that re-entrant processing is a mechanism that is generally at play when emotional stimuli, being biological or culturally acquired, have to be processed.

ACKNOWLEDGMENTS

We are very grateful to Andreas Wollbrink, Bertram Walter, and Max Bruchmann for their valuable advice and comments.

[3]It should be noted that effect sizes and extensions of significant clusters are independent of the nonuniqueness of the inverse solution, i.e., the estimated localisation of a strong effect is not necessarily more reliable than the localization of a weaker effect. The resolution of EEG and especially of MEG is optimal for superficial neural generators but deteriorates with increasing depth. Depth weighting, as performed in this study, expands the space of possible source locations, which in turn increases the number of possible solutions (i.e., nonuniqueness of the inverse solution). The combination of EEG and MEG can partly compensate for this problem and is specifically important, when participation of deep brain structures is expected. However, due to the nonuniqueness of the inverse solution, the localization-especially of deep clusters-needs to be interpreted with caution. Therefore, the finding of PCC involvement in the generation of the EPN requires replication, for example by means of a combined EEG-fMRI study.

REFERENCES

Anderson AK, Phelps EA (2001): Lesions of the human amygdala impair enhanced perception of emotionally salient events. Nature 411:305–309.

Assadollahi R, Pulvermüller F (2003): Early influences of word length and frequency: A group study using MEG. Neuroreport 14:1183–1187.

Baayen R, Piepenbrock R, Gulikers L (1995): The CELEX lexical database (Release 2). Linguistic Data Consortium.Philadelphia, PA:University of Pennsylvania.

Bechara A, Tranel D, Damasio H, Adolphs R (1995): Double dissociation of conditioning and declarative knowledge relative to the amygdala and hippocampus in humans. Science 269: 1115–1118.

Bradley MM, Lang PJ (1994): Measuring emotion: The self-assessment manikin and the semantic differential. J Behav Ther Exp Psychiatry 25:49–59.

Bradley MM, Sabatinelli D, Lang PJ, Fitzsimmons JR, King W, Desai P (2003): Activation of the visual cortex in motivated attention. Behav Neurosci 117:369–380.

Bruchmann M, Herper K, Konrad C, Pantev C, Huster RJ (2010): Individualized EEG source reconstruction of stroop interference with masked color words. Neuroimage 49:1800–1809.

Cato MA, Crosson B, Gökçay D, Soltysik D, Wierenga C, Gopinath K, Himes N, Belanger H, Bauer RM, Fischler IS, Gonzalez-Rothi L, Briggs RW (2004): Processing words with emotional connotation: An fMRI study of time course and laterality in rostral frontal and retroplenial cortices. J Cogn Neurosci 16:167–177.

Cohen D, Cuffin BN (1983): Demonstration of useful differences between magnetoencephalogram and electroencephalogram. Electroencephalogr Clin Neurophysiol 56:38–51.

Cox RW (1996): AFNI software for analysis and visualization of functional magnetic resonance neuroimages. Comput Biomed Res 29:162–173.

Culham JC, Kanwisher NG (2001): Neuroimaging of cognitive functions in human parietal cortex. Curr Opin Neurobiol 11:157–163.

Damasio AR (1996): The somatic marker hypothesis and the possible functions of the prefrontal cortex. Philos Trans R Soc Lond B Biol Sci 351:1413–20.

Dobel C, Junghöfer M, Breitenstein C, Klauke B, Knecht S, Pantev C, Zwitserlood P (2010): New names for known things: On the association of novel word forms with existing semantic information. J Cogn Neurosci 22:1251–1261.

Dolcos F, LaBar KS, Cabeza R (2004): Dissociable effects of arousal and valence on prefrontal activity indexing emotional evaluation and subsequent memory: An event-related fMRI study. Neuroimage 23:64–74.

Dronkers NF, Wilkins DP, Van Valin RD Jr, Redfern BB, Jaeger JJ (2004): Lesion analysis of the brain areas involved in language comprehension. Cognition 92:145–177.

Flaisch T, Häcker F, Renner B, Schupp HT (2011): Emotion and the processing of symbolic gestures: An event-related brain potential study. Soc Cogn Affect Neurosci 6:109–118.

Freese JL, Amaral DG (2005): The organization of projections from the amygdala to visual cortical areas TE and V1 in the macaque monkey. J Comp Neurol 486:295–317.

Fuchs M, Wagner M, Wischmann H-A, Köhler T, Theissen A, Drenckhahn R, Buchner H (1998): Improving source reconstructions by combining bioelectric and biomagnetic data. Electroencephalogr Clin Neurophysiol 107:93–111.

Fuchs M, Wagner M, Köhler T, Wischmann H-A (1999): Linear and nonlinear current density reconstructions. J Clin Neurophysiol 16:267–295.

Fuchs M, Kastner J, Wagner M, Hawes S, Ebersole J (2002): A standardized boundary element method volume conductor model. Clin Neurophysiol 113:702–712.

Geddes LA, Baker LE (1963): The specific resistance of biological material, a compendium of data for the biomedical engineer and physiologist. Med Biol Eng 5:271–293.

Ghashghaei HT, Barbas H (2002): Pathways for emotion: Interactions of prefrontal and anterior temporal pathways in the amygdala of the rhesus monkey. Neuroscience 115:1261–1279.

Goldenholz DM, Ahlfors SP, Hämäläinen MS, Sharon D, Ishitobi M, Vaina LM, Shufflebeam SM (2009): Mapping the signal-to-noise-ratios of cortical sources in magnetoencephalography and electroencephalography. Hum Brain Mapp 30:1077–1086.

Gothard KM, Battaglia FP, Erickson CA, Spitler KM, Amaral DG (2007): Neural responses to facial expression and face identity in the monkey amygdala. J Neurophysiol 97:1671–1683.

Hauk O, Davis MH, Ford M, Pulvermüller F, Marslen-Wilson WD (2006): The time course of visual word recognition as revealed by linear regression analysis of ERP data. Neuroimage 30:1383–1400.

Hauk O, Pulvermüller F, Ford M, Marslen-Wilson WD, Davis MH (2009): Can I have a quick word? Early electrophysiological manifestations of psycholinguistic processes revealed by event-related regression analysis of the EEG. Biol Psychol 80:64–74.

Hauk O, Wakeman DG, Henson R (2011): Comparison of noise-normalized minimum norm estimates for MEG analysis using multiple resolution metrics. Neuroimage 54:1966–1974.

Hauk O, Coutout C, Holden A, Chen Y (2012): The time-course of single-word reading: Evidence from fast behavioral and brain responses. Neuroimage 60:1462–1477.

Herbert C, Junghöfer M, Kissler J (2008): Event related potentials to emotional adjectives during reading. Psychophysiology 45:487–498.

Herbert C, Ethofer T, Anders S, Junghofer M, Wildgruber D, Grodd W, Kissler J (2009): Amygdala activation during reading of emotional adjectives—An advantage for pleasant content. Soc Cogn Affect Neurosci 4:35–49.

Hirschfeld G, Zwitserlood P, Dobel C (2011): Effects of language comprehension on visual processing—MEG dissociates early perceptual and late N400 effects. Brain Lang 116:91–96.

Hofmann MJ, Kuchinke L, Tamm S, Võ ML-H, Jacobs AM (2009): Affective processing within 1/10th of a second: High arousal is necessary for early facilitative processing of negative but not positive words. Cogn Affect Behav Neurosci 9:389–397.

Hämäläinen MS, Hari R, Ilmoniemi RJ, Knuutila J, Lounasmaa OV (1993): Magnetoencephalography—Theory, instrumentation, and application to noninvasive studies of the working human brain. Rev Mod Phys 65:413–497.

Hämäläinen MS, Ilmoniemi RJ (1994): Interpreting magnetic fields of the brain: Minimum norm estimates. Med Biol Eng Comput 32:35–42.

Ille N, Berg P, Scherg M (2002): Artifact correction of the ongoing EEG using spatial filters based on artifact and brain signal topographies. J Clin Neurophysiol 19:113–124.

Indefrey P, Cuttler A (2004): Prelexical and lexical processing in listening. In: Gazzaniga MS, editor. The Cognitive Neurosciences.Cambridge, MA:MIT Press. pp759–774.

Indefrey P, Levelt WJM (2004): The spatial and temporal signatures of word production components. Cognition 92:101–144.

Ioannides AA, Liu MJ, Liu LC, Bamidis PD, Hellstrand E, Stephan KM (1995): Magnetic field tomography of cortical and deep processes: Examples of "real-time mapping" of averaged and single trial MEG signals. Int J Psychophysiol 20:161–175.

Isenberg N, Silbersweig D, Engelien A, Emmerich S, Malavade K, Beattie B, Leon A, Stern E (1999): Linguistic threat activates the human amygdala. Proc Natl Acad Sci 96:10456–10456.

Junghöfer M, Bradley MM, Elbert TR, Lang PJ (2001): Fleeting images: A new look at early emotion discrimination. Psychophysiol 38:175–178.

Junghöfer M, Peyk P, Flaisch T, Schupp HT (2006): Neuroimaging methods in affective neuroscience: Selected methodological issues. Prog Brain Res 156:123–143.

Keil A, Sabatinelli D, Ding M, Lang PJ, Ihssen N, Heim S (2009): Re-entrant projections modulate visual cortex in affective perception: Evidence from Granger causality analysis. Hum Brain Mapp 30:532–540.

Kensinger EA, Corkin S (2004): Two routes to emotional memory: Distinct neural processes for valence and arousal. Proc Natl Acad Sci 101:3310–3315.

Kissler J, Assadollahi R, Herbert C (2006): Emotional and semantic networks in visual word processing: Insights from ERP studies. Prog Brain Res 156:147–183.

Kissler J, Herbert C, Peyk P, Junghöfer M (2007): Buzzwords: Early cortical responses to emotional words during reading. Psychol Sci 18:475–480.

Kissler J, Herbert C, Winkler I, Junghofer M (2009): Emotion and attention in visual word processing: An ERP study. Biol Psychol 80:75–83.

Krolak-Salmon P, Henaff MA, Vighetto A, Bertrand O, Mauguiere F (2004): Early amygdala reaction to fear spreading in occipital, temporal, and frontal cortex: A depth electrode ERP study in human. Neuron 42:665–676.

Kuchinke L, Jacobs AM, Grubich C, Võ M L-H, Conrad M, Herrmann M (2005): Incidental effects of emotional valence in single word processing: An fMRI study. Neuroimage 28:1022–1032.

Kutas M, Federmeier KD (2000): Electrophysiology reveals semantic memory use in language comprehension. Trends Cogn Sci 4:463–470.

Laeger I, Dobel C, Dannlowski U, Kugel H, Grotegerd D, Kissler J, Keuper K, Eden A, Zwitserlood P, Zwanzger P (2012): Amygdala responsiveness to emotional words is modulated by subclinical anxiety and depression. Behav Brain Res 233:508–516.

Lang PJ, Bradley MM, Cuthbert BN (1997): Motivated attention: Affect, activation, and action. In: Lang PJ, Simons RF, Balaban M, editors. Attention and Emotion: Sensory and Motivational Processes.New York:Erlbaum. pp97–135.

Lau E, Phillips C, Poeppel D (2008): A cortical network for semantics: (de)constructing the N400. Nat Rev Neurosci 9:920–33.

LeDoux JE (2000): Emotion circuits in the brain. Annu Rev Neurosci 23:155–184.

Lewis PA, Critchley HD, Rotshtein P, Dolan RJ (2007): Neural correlates of processing valence and arousal in affective words. Cereb cortex 17:742–748.

Lin FH, Witzel T, Ahlfors SP, Stufflebeam SM, Belliveau JW, Hamalainen MS (2006): Assessing and improving the spatial accuracy in MEG source localization by depth-weighted minimum-norm estimates. Neuroimage 31:160–171.

Luo Q, Holroyd T, Jones M, Hendler T, Blair J (2007): Neural dynamics for facial threat processing as revealed by gamma band synchronization using MEG. Neuroimage 34:839–847.

Luo Q, Mitchell D, Cheng X, Mondillo K, McCaffrey D, Holroyd T, Carver F, Coppola R, Blair J (2009): Visual awareness, emotion, and gamma band synchronization. Cereb Cortex 19:1896–1904.

Maddock RJ (1999): The retrosplenial cortex and emotion: New insights from functional neuroimaging of the human brain. Trends Neurosci 22:310–316.

Maddock RJ, Garrett AS, Buonocore MH (2003): Posterior cingulate cortex activation by emotional words: fMRI evidence from a valence decision task. Hum Brain Mapp 18:30–41.

Molins A, Stufflebeam SM, Brown EN, Hämäläinen MS (2008): Quantification of the benefit from integrating MEG and EEG data in minimum l2-norm estimation. Neuroimage 42:1069–1077.

Montoya P, Larbig W, Pulvermüller F, Flor H, Birbaumer N (1996): Cortical correlates of semantic classical conditioning. Psychophysiol 33:644–649.

Mormann F, Kornblith S, Quiroga RQ, Kraskov A, Cerf M, Fried I, Koch C (2008): Latency and selectivity of single neurons indicate hierarchical processing in the human medial temporal lobe. J Neurosci 28:8865–8872.

Morris JS, Friston KJ, Büchel C, Frith CD, Young AW, Calder AJ, Dolan RJ (1998): A neuromodulatory role for the human amygdala in processing emotional expressions. Brain 121:47–57.

Morris JS, Ohman A, Dolan R (1999): A subcortical pathway to the right amygdala mediating 'unseen' fear. Proc Natl Acad Sci 96:1680–1685.

Mouchetant-Rostaing Y, Giard MH, Delpuech C, Echallier JF, Pernier J (2000): Early signs of visual categorization for biological and non-biological stimuli in humans. Neuroreport 11:2521–2525.

Olofsson JK, Nordin S, Sequeira H, Polich J (2008): Affective picture processing: An integrative review of ERP findings. Biol Psychol 77:247–265.

Ortigue S, Michel CM, Murray MM, Mohr C, Carbonnel S, Landis T (2004): Electrical neuroimaging reveals early generator modulation to emotional words. Neuroimage 21:1242–1251.

Oya H, Kawasaki H, Howard MA, Adolphs R (2002): Electrophysiological responses in the human amygdala discriminate emotion categories of complex visual stimuli. J Neurosci 22:9502–9512.

Palazova M, Mantwill K, Sommer W, Schacht A (2011): Are effects of emotion in single words non-lexical? Evidence from event-related brain potentials. Neuropsychologia 49:2766–2775.

Pessoa L, Adolphs R (2010): Emotion processing and the amygdala: From a low road' to 'many roads' of evaluating biological significance. Nat Rev Neurosci 11:773–783.

Peyk P, Schupp HT, Elbert T, Junghöfer M (2008): Emotion processing in the visual brain: A MEG analysis. Brain Topogr 20:205–215.

Peyk P, De Cesarei A, Junghöfer M (2011): ElectroMagnetoEncephaloGraphy Software: Overview and integration with other EEG/MEG toolboxes. Comput Intell Neurosci 2011. doi:10.1155/2011/861705.

Poeppel D, Hickok G (2004): Towards a new functional anatomy of language. Cognition 92:1–12.

Price CJ (2000): The anatomy of language: Contributions from functional neuroimaging. J Anat 197:335–359.

Price CJ (2010): The anatomy of language: A review of 100 fMRI studies published in 2009. Ann N Y Acad Sci 1191:62–88.

Pulvermüller F (2001): Brain reflections of words and their meaning. Trends Cogn Sci 5:517–524.

Pulvermüller F, Shtyrov Y, Hauk O (2009): Understanding in an instant: Neurophysiological evidence for mechanistic language circuits in the brain. Brain Lang 110:81–94.

Rämä P, Martinkauppi S, Linnankoski I, Koivisto J, Aronen HJ, Carlson S (2001): Working memory of identification of emotional vocal expressions: An fMRI study. Neuroimage 13:1090–1101.

Sabatinelli D, Bradley MM, Fitzsimmons JR, Lang PJ (2005): Parallel amygdala and inferotemporal activation reflect emotional intensity and fear relevance. Neuroimage 24:1265–1270.

Salmelin R, Baillet S (2009): Electromagnetic brain imaging. Hum Brain Mapp 30:1753–1757.

Schacht A, Sommer W (2009a): Emotions in word and face processing: Early and late cortical responses. Brain Cogn 69:538–550.

Schacht A, Sommer W (2009b): Time course and task dependence of emotion effects in word processing. Cogn Affect Behav Neurosci 9:28–43.

Schupp HT, Junghöfer M, Weike AI, Hamm AO (2004a): The selective processing of briefly presented affective pictures: An ERP analysis. Psychophysiol 41:441–449.

Schupp HT, Öhman A, Junghöfer M, Weike AI, Stockburger J, Hamm AO (2004b): The facilitated processing of threatening faces: An ERP analysis. Emotion 4:189–200.

Scott GG, O'Donnell PJ, Leuthold H, Sereno SC (2009): Early emotion word processing: Evidence from event-related potentials. Biol psychol 80:95–104.

Sereno SC, Rayner K, Posner MI (1998): Establishing a time-line of word recognition: Evidence from eye movements and event-related potentials. Neuroreport 9:2195–2200.

Sereno SC, Rayner K (2003): Measuring word recognition in reading: Eye movements and event-related potentials. Trends Cogn Sci 7:489–493.

Sharon D, Haämäläinen MS, Tootell RB, Halgren E, Belliveau JW (2007): The advantage of combining MEG and EEG: Comparison to fMRI in focally stimulated visual cortex. Neuroimage 36:1225–1235.

Skrandies W (1998): Evoked potential correlates of semantic meaning—A brain mapping study. Brain Res Cogn Brain Res 6:173–183.

Song XW, Dong ZY, Long XY, Li SF, Zuo XN, Zhu CZ, He Y, Yan CG, Zang YF (2011): REST: a toolkit for resting-state functional magnetic resonance imaging data processing. PLoS ONE 6:1–12.

Steinberg C, Dobel C, Schupp HT, Kissler J, Elling L, Pantev C, Junghöfer M (2012): Rapid and highly resolving: Affective evaluation of olfactorily conditioned faces. J Cogn Neurosci 24:17–27.

Steinberg C, Bröckelmann AK, Rehbein M, Dobel C, Junghöfer M (2012): Rapid and highly resolving associative affective learning: Convergent electro- and magnetoencephalographic evidence from vision and audition. Biol Psychol. doi:10.1016/j.biopsycho.2012.02.009.

Straube T, Sauer A, Miltner WHR (2011): Brain activation during direct and indirect processing of positive and negative words. Behav Brain Res 222:66–72.

Streit M (2003): Time course of regional brain activations during facial emotion recognition in humans. Neurosci Lett 342:101–104.

Tabert MH, Borod JC, Tang CY, Lange G, Wei TC, Johnson R, Nusbaum AO, Buchsbaum MS (2001): Differential amygdala activation during emotional decision and recognition memory tasks using unpleasant words: An fMRI study. Neuropsychologia 39:556–573.

Vuilleumier P (2005): How brains beware: Neural mechanisms of emotional attention. Trends Cogn Sci 9:585–594.

Vuilleumier P, Huang Y-M (2009): Emotional attention: Uncovering the mechanisms of affective biases in perception. Curr Dir Psychol Sci 18:148–152.

Wolters CH, Anwander A, Tricoche X, Weinstein D, Koch MA, MacLeod RS (2006): Influence of tissue conductivity anisotropy on EEG/MEG field and return current computation in a realistic head model: A simulation and visualization study using high-resolution finite element modeling. Neuroimage 30:813–826.

Zschocke S (2009): Klinische Elektroenzephalographie. Berlin: Springer.

Zwitserlood P (1989): The locus of the effects of sentential-semantic context in spoken-word processing. Cognition 32:25–64.
